# The Applied Sports Science and Medicine of Netball: A Systematic Scoping Review

**DOI:** 10.1007/s40279-021-01461-6

**Published:** 2021-06-04

**Authors:** Sarah Whitehead, Jonathon Weakley, Stuart Cormack, Helen Alfano, Jim Kerss, Mitch Mooney, Ben Jones

**Affiliations:** 1 Carnegie Applied Rugby Research (CARR) Centre, Carnegie School of Sport, Leeds Beckett University, Leeds, LS6 3QS UK; 2 Leeds Rhinos Netball, Leeds, UK; 3 Leeds Rhinos Rugby League Club, Leeds, UK; 4 School of Behavioural and Health Sciences, Australian Catholic University, Brisbane, QLD Australia; 5 School of Behavioral and Health Sciences, Australian Catholic University, Melbourne, VIC Australia; 6 Sports Performance, Recovery, Injury and New Technologies (SPRINT) Research Centre, Australian Catholic University, Melbourne, Australia; 7 England Netball, Loughborough, UK; 8 English Institute of Sport, Manchester, UK; 9 High Performance Pillar, Netball Australia, Melbourne, Australia; 10 England Performance Unit, The Rugby Football League, Leeds, UK; 11 School of Science and Technology, University of New England, Armidale, NSW Australia; 12 Division of Exercise Science and Sports Medicine, Department of Human Biology, Faculty of Health Sciences, The University of Cape Town and the Sports Science Institute of South Africa, Cape Town, South Africa

## Abstract

**Background:**

Netball is the one of the most popular women’s sports in the world. Since gaining professional status in 2008 there has been a rapid growth in research in the applied sports science and medicine of the sport. A scoping review of the area would provide practitioners and researchers with an overview of the current scientific literature to support on-court performance, player welfare and reduce injury.

**Objective:**

The primary objective was to identify the current research on the applied sports science and medicine of netball. Additionally, the article provides a brief summary of the research in each topic of sports science and medicine in netball and identifies gaps in the current research.

**Methods:**

Systematic searches of PubMed, SPORTDiscus, MEDLINE and CINAHL were undertaken from earliest record to Dec 2020 and reference lists were manually searched. The PRISMA-ScR protocol was followed. Studies were eligible for inclusion if they investigated netball as a sport or the applied sport science and medicine of netball athletes.

**Results:**

962 studies were identified in the initial search, 150 of which met the inclusion criteria. Injury was the most highly investigated sport science and medicine topic (*n* = 45), followed by physical qualities (*n* = 37), match characteristics (*n* = 24), biomechanics (*n* = 15), psychology (*n* = 13), fatigue and recovery (*n* = 9), training load (*n* = 4) and nutrition (*n* = 3). A range of cohorts were used from school to elite and international standards. All cohorts were female netballers, except for one study. A rapid growth in studies over recent years was demonstrated with 65% of studies published in the last decade. There still remains gaps in the literature, with a low evidence base for nutrition, training load and fatigue and recovery.

**Conclusion:**

This scoping review summarises the current evidence base and key findings that can be used in practice to enhance the applied sport science and medical support to netball athletes across a range of playing standards, and support the growth of the sport. It is evident that netball as a sport is still under-researched.

**Supplementary Information:**

The online version contains supplementary material available at 10.1007/s40279-021-01461-6.

## Key Points

**Table:** 

Research into the applied sports science and medicine of netball has increased substantially over the past decade, with the majority of research originating from Australia. The growth in research aligns with the professionalisation of the sport and the development of technology.
Injury is the most commonly researched topic, and a systematic review and meta-analysis is required in this topic to provide researchers and practitioners with a high level of evidence regarding incidence and prevalence, in addition to injury mechanism and site. Physical qualities follows as the second most researched topic, with studies focusing on testing, training interventions and reporting the physical characteristics in a range of netball athletes.
Priority topics for future research include: the burden and recurrence of injuries, the role of physical qualities and contextual factors on injury and performance, and quantification and appropriateness of training load across elite and age-grade netball.

## Introduction

Netball is one of the most popular women’s sports in the world, with 20 million participants across 80 countries, and 70 National Netball Associations affiliated to the International Netball Federation (INF) across Africa, Asia, America, Europe and Oceania [1]. In 1995 it became a ‘recognised’ sport of the International Olympic Committee, and in 1998 was included in the Commonwealth Games programme for the first time [2]. In 2008, netball had its first semi-professional league formed (ANZ Championship), with professional or semi-professional leagues now in Australia (Suncorp Super Netball), New Zealand (ANZ Premiership), the United Kingdom (UK; Vitality Netball Superleague) and South Africa (Telkom Netball League). As the sport has transitioned to professional status, there has been an increase in research across a range of sport science and medicine areas [3–10]. Consequently, a scoping review of the area would provide practitioners and researchers with an overview of the current scientific literature to support on-court performance and reduce injury.

Netball is a dynamic, high-intensity, intermittent court-based team sport [1, 11–13]. It is played over 60-min split into 15-min quarters, with 4-min breaks between quarters 1–2 and 3–4 and an 8–12-min half-time break between quarters 2–3, in professional leagues [11, 14, 15]. The traditional game of netball is played with seven positions: goal shooter (GS), goal attack (GA), wing attack (WA), center (C), wing defence (WD), goal defence (GD) and goal keeper (GK), on a 15.25 × 30.50 m court divided into thirds [16, 17]. A five-a-side version has also gained popularity in recent years with the Fast5 Netball World series, which is played on the same court dimensions but has six-minute quarters [17]. The two teams competing in a game of netball strive to keep or gain possession of the ball and score a goal by shooting the ball through a ring that is 3.05 m high [17]. The rules restrict players to: playing position specified areas of the court, moving only one step when in possession of the ball, and releasing the ball within three seconds of receiving it [17]. Rule changes in 2016 were designed to increase the match speed by providing playing ‘advantage’ and reducing the number of timeouts [18]. Such rules and the discrete skill set required [19], demand a unique set of physical qualities and mental skills from the players, particularly at the elite level [18, 20–22].

Over the last 30 years research into the applied sport science and medicine of team sports has grown rapidly (e.g., rugby league [23, 24] Australian Rules [25, 26]). Specifically, recent increases have been observed in netball, likely due to the professionalisation and popularity of the sport as well as the development in technologies. Netball has unique physical (e.g., agility maneuvers, jumping, landing [12, 27]), technical (e.g., guarding, passing, and shooting [19, 28]) and tactical (e.g., set-plays [29]) requirements, which necessitates evidence-based support through sport science and medical provision. There is a high injury rate of netball players within the community [30, 31] (e.g., 14 injuries per 1000 h [31]) and elite game [32, 33] (e.g., up to ~ 500 injuries per 1000 h in elite South African players [33]), leading to the implementation of injury reduction strategies [34, 35]. The physical qualities of players and activity profiles of match-play have also been quantified to aid in development and preparation for performance [11, 12, 15, 16, 18]. However, whilst practical recommendations for strength and conditioning coaches working with netball athletes have been provided [36–38], a review of the current applied sport science and medicine of netball literature following a systematic approach is yet to be carried out.

The purpose of this scoping review is to provide an overview of existing research on the applied sport science and medicine of netball. Four primary objectives were to: (1) conduct a systematic search of the published literature, (2) map out the characteristics of the research, (3) provide a brief summary of the research in each area of sport science and medicine, and (4) identify gaps in the current research.

## Methods

### Design and Search Strategy

A scoping review was carried out in accordance with the Preferred Reporting Items for Systematic reviews and Meta-analyses extension for Scoping Reviews (PRISMA-ScR) [39]. A systematic search of electronic databases (PubMed, SPORTDiscus, MEDLINE and CINAHL) was performed from the earliest record to 28th Dec 2020. All study designs were included. The search strategy combined the term ‘netball’ AND terms covering topics of the applied sport science and medicine of netball: ‘demands’ OR ‘characteristics’ OR ‘match-play’ OR ‘matches’ OR ‘match’ OR ‘game’ OR ‘game-play’ OR ‘competition’ OR ‘performance’ OR ‘skill’ OR ‘technical’ OR ‘physical’ OR ‘testing’ OR ‘qualities’ OR ‘anthropometrics’ OR ‘composition’ OR ‘strength’ OR ‘speed’ OR ‘power’ OR ‘fitness’ OR ‘change of direction’ OR ‘agility’ OR ‘jump’ OR ‘physiology’ OR ‘training’ OR ‘load’ OR ‘exposure’ OR ‘fatigue’ OR ‘recovery’ OR ‘muscle damage’ OR ‘development’ OR ‘intervention’ OR ‘injury’ OR ‘wellness’ OR ‘wellbeing’ OR ‘risk’ OR ‘incidence’ OR ‘health’ OR ‘psychology’ OR ‘mental’. Reference lists of selected papers were manually searched for other potentially eligible papers.

### Study Selection

After eliminating duplicates, search results were screened independently by two researchers (SW, JW) against the eligibility criteria. Disagreements were resolved through discussion or via a third researcher (BJ). Articles which could not be eliminated by the title or abstract were retrieved and evaluated for inclusion via a full-text review. The title and authors were not masked to the reviewers.

Studies were eligible for inclusion if they investigated netball as a sport or the applied sport science of netball athletes from a ‘performance perspective’, or investigated injury epidemiology with outcome measures reported for netball athletes. Only original research investigations in peer-reviewed journals were included.

Studies were excluded from the review if they did not investigate netball, or they used netball athletes as subjects but did not investigate netball as a sport or the applied sport science and medicine of netball athletes. For example, Ashton and Twist [40] used university netball players as subjects but the purpose was to investigate the impact of change of direction (CoD) on the physiological responses during generic shuttle running and the study was therefore excluded. Such studies that use netball athletes as subjects to examine a broader concept have limited direct application to impact upon netball specifically. Studies that investigated netball umpires only or that examined netball from a coaching or physical activity/participation perspective were excluded. Review articles and conference proceedings with abstracts only were excluded. Papers from all languages were included but excluded if translation to English could not be made. When authors could not be contacted to retrieve full texts, studies were excluded.

### Data Extraction

Authors (SW, JW, BJ) reviewed the studies and discussed the overarching sport science and medicine topics. Studies were categorised into these topics, determined by their primary aims and outcome measures, with sub-categories identified where appropriate. The general characteristics (i.e., year of publication, geography, cohort investigated, sample size) of each study were extracted. Data relating to the participants’ characteristics (i.e., sex, age, stature, body mass), the aim, outcome measures, and key findings of each study relating to the purpose of this review were extracted. Outcome measures were converted into comparable units, e.g., stature converted from m to cm. Where necessary, means and measures of dispersion were extracted from figures in the manuscripts using WebPlotDigitizer v4.2 [41].

### Data Synthesis

Given the purpose of a scoping review is to first map the extent, range, and nature of the literature on a topic, and secondly, summarise findings that are heterogeneous [39], no analysis was carried out. Study characteristics, key outcomes, and data are summarised with data presented as mean ± standard deviation (SD) where appropriate.

## Results and Discussion

### Search and Selection of Studies

**Fig. 1:**
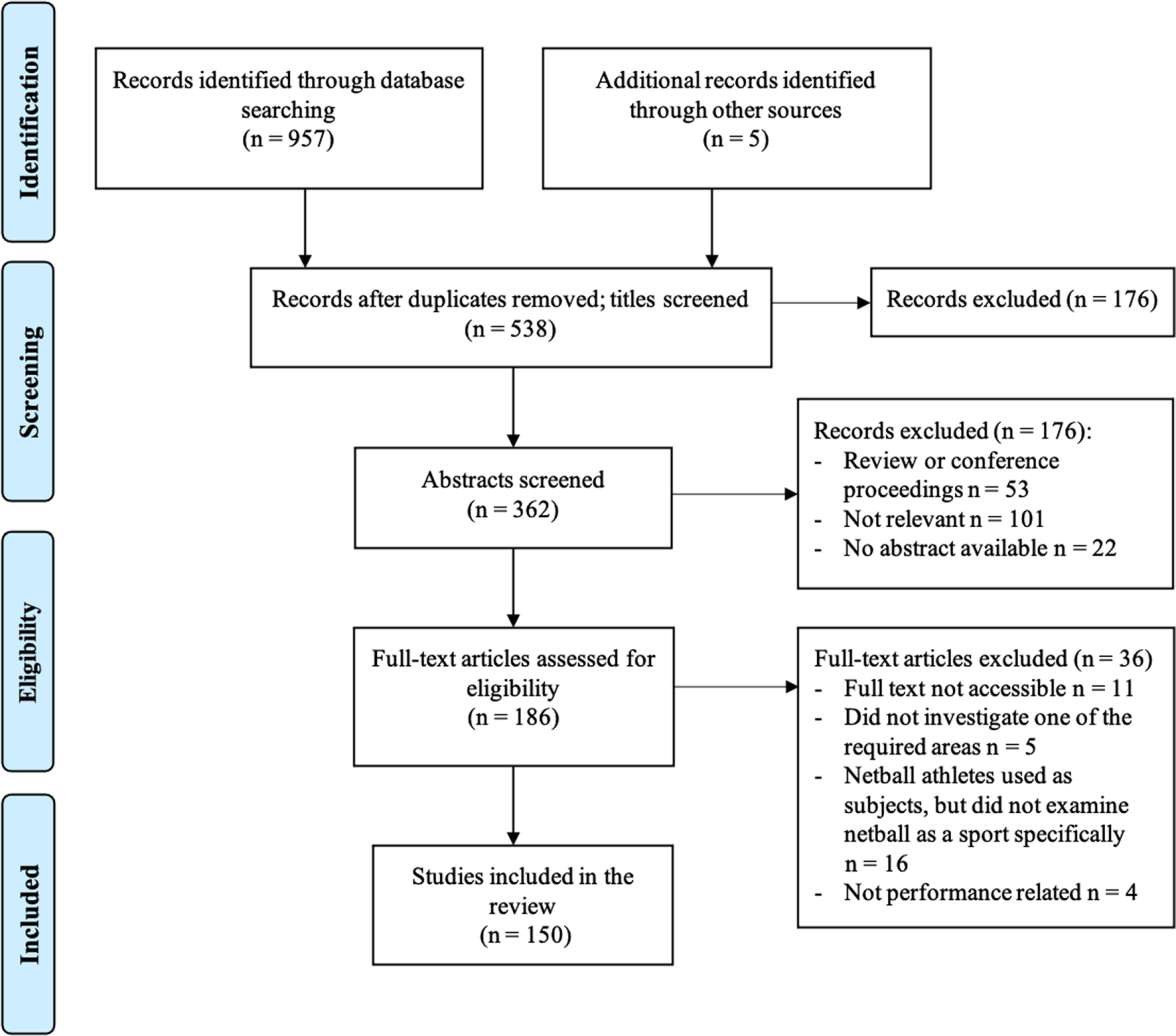
Flow of selection process for eligible studies for inclusion

The database search identified 957 articles, with five studies identified through other sources. Following the removal of duplicates and screening for eligibility, 150 studies remained for inclusion in the review. The flow of articles through identification to final inclusion is shown in Fig. 1.

### General Characteristics of the Studies

#### Sports Science and Medicine Topics

**Fig. 2:**
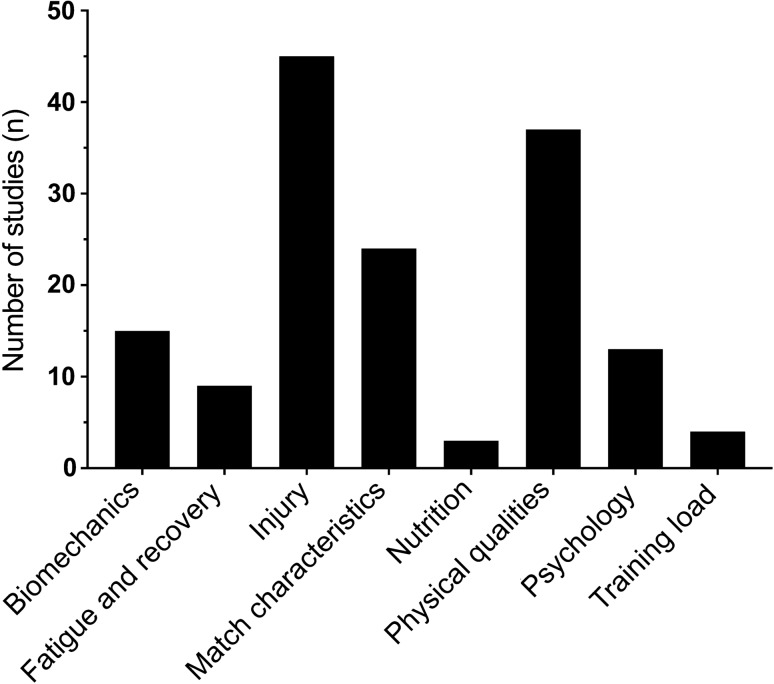
Netball sport science and medicine topics of included studies

The 150 studies included in the review covered eight sport science and medicine topics: biomechanics (*n* = 15, 10%), fatigue and recovery (*n* = 9, 6%), injury (*n* = 45, 30%), match characteristics (*n* = 24, 16%), nutrition (*n* = 3, 2%), physical qualities (*n* = 37, 25%), psychology (*n* = 13, 9%), and training load (*n* = 4, 3%) (Fig. 2).

#### Publication Year

Table 1 demonstrates the rapid growth in studies over recent years, with 65% of studies published between 2011 and 2020. Studies investigating injury range from one study before 1990 (2%) [42], to 22 studies between 2011 and 2020 (49%). All other topics had over 60% of studies published after 2010. The growth in studies coincides with the increased professionalism of netball and thus the potential increase in funding for research. Additionally, improvements in technology (e.g., inertial movement units) have enhanced the ability to quantify (e.g., external workload) team sports [43, 44], which would explain the increase in match-characteristics research.

**Table 1:** The year of publication of studies included in the review

Research topic	Year group published							
	Before 1990		1990–2000		2001–2010		2011–April 2020	
	Number of studies	% of studies	Number of studies	% of studies	Number of studies	% of studies	Number of studies	% of studies
Biomechanics	1	7	1	7	3	20	10	67
Fatigue and recovery	0	0	0	0	1	11	8	89
Injury	1	2	10	22	12	27	22	49
Match characteristics	0	0	1	4	4	17	19	79
Nutrition	0	0	1	33	0	0	2	67
Physical qualities	1	3	1	3	10	27	25	68
Psychology	0	0	3	23	2	15	8	62
Training load	0	0	0	0	0	0	4	100
Total	3	2	17	11	32	21	98	65

#### Geography of studies

**Fig. 3:**
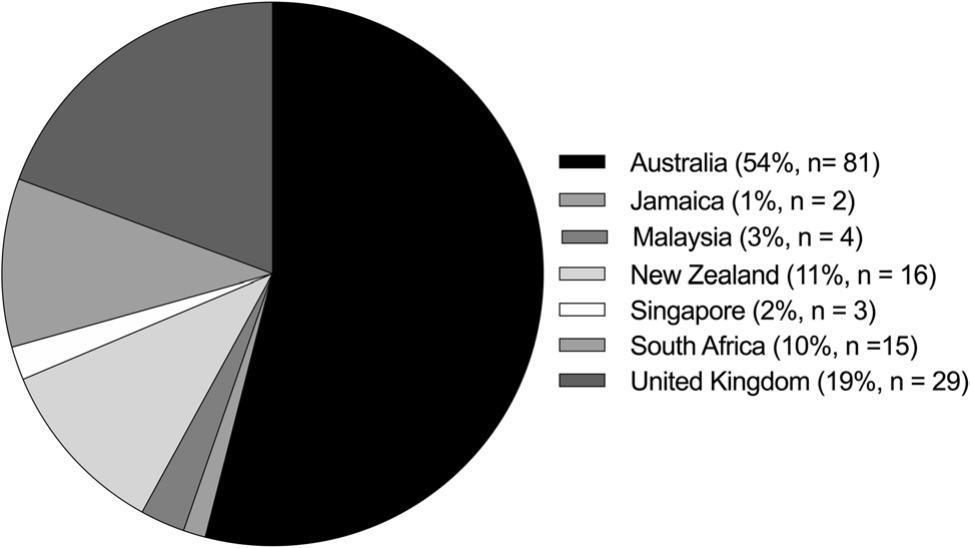
The geography of included studies

Studies were identified from seven different countries: Australia, Jamaica, Malaysia, New Zealand, Singapore, South Africa and the UK (Fig. 3). The majority of studies were from Australia (*n* = 81), followed by the UK (*n* = 29) and New Zealand (*n* = 16) (Fig. 3), which have high participation rates and semi-/professional leagues. Within the topic of ‘match-characteristics’, 20 out of the 24 studies (83%) were from Australia, which is unsurprising given Australia has the most professional (in terms of funding and spectators) netball competition (Suncorp Super Netball). Nutrition and psychology were the only topics in which studies from Australia did not dominate with 67% (*n* = 2) and 54% (*n* = 7) from the UK, but with both still being under-investigated relative to other topics (Fig. 2). The countries of very low representation (Malaysia [*n* = 4], Singapore [*n* = 3], Jamaica [*n* = 2]) investigate injury [32, 45–47], physical qualities [48–51] and biomechanics only [52].

### Biomechanics

Fifteen studies investigated biomechanical outcomes related to netball (Supplementary Table S1). Studies used a range of athletes from recreationally trained (*n* = 3, 20%) to elite level (*n* = 4, 27%). The majority of studies (80%, *n* = 12) investigated biomechanical outcomes related to injury risk [8–10, 52–60]. Movement analysis of the shoulder pass [19] and shooting [61] have also been carried out, and the influence of playing surface on landing has been assessed [62]. Differences in shooting action between age-groups have been investigated [61]. Senior-level players extend their elbows and knees simultaneously, while juniors appear to have greater dissociation between the upper and lower peripheries [61]. Furthermore, senior-level players have greater variability of movement between their upper limbs when shooting [61]. A case study (*n* = 1) identified that during the shoulder pass, the greatest ground reaction forces (GRF) have been observed to coincide with ball release [19]. However, it is unknown whether differences in experience and passing success elicit different passing strategies.

#### Injury Risk

During netball-related landing tasks, well-trained players demonstrated substantial valgus of the knee [10], with Collings et al. [53] demonstrating that experience level may not mitigate risk factors associated with poor frontal plane knee control. However, empirical evidence is still required to better understand whether differences in landing technique can modify injury risk.

Seven studies [8, 9, 54, 56, 58, 60] investigated the use of strapping and bracing of joints collectively demonstrating that strapping and bracing may reduce the range of motion but do not influence joint kinetics or kinematics. Yet, there is evidence to suggest that proprioception is improved with ankle bracing [59] and athletes perceive greater stability in the strapped or braced joint [9, 58]. Furthermore, whilst the joints may not be affected when landing, it is possible that muscle activity is reduced [8]. This information suggests that bracing and taping joints may provide the athlete a perceived benefit rather than actual changes in the kinetic and kinematic outputs and whether these practices cause worthwhile reductions in injury risk is still unknown. Additionally, further investigation is required on whether lower limb movement screening [55] can help identify future injury risk in netball athletes.

### Fatigue and Recovery

Nine studies investigated fatigue and recovery within netball (Supplementary Table S2). The majority of studies used elite level cohorts (*n* = 7, 78%) [63–69], with the remaining studies using Australian state-level athletes [3, 70]. Two studies (22%) quantified the fatigue response to competition [63, 67], whilst two studies (22%) investigated the effect [64] and the perceived importance [68] of various recovery modalities. Four studies (44%) focused on sleep indices, patterns and/or behaviours [3, 65, 66, 69]. Finally, one study investigated the influence of compression garments on netball-specific running performance during a circuit [70].

Studies investigating the fatigue responses have shown a varying time-course of recovery at 62 h post-match (i.e., not all variables returned to baseline) over a 3-day international tournament [63] and that subjective mental and physical fatigue were reported to be separate constructs, with limited relationships with performance variables following matches [67]. Sleep and fluid replacements were reported as the perceived most important recovery modalities in 215 netball players [68]. Additionally, Juliff et al. [64] found that following a netball-specific circuit contrast water therapy and contrast showers improved perception of recovery in comparison to the passive recovery condition, but no difference in physical recovery was reported. However, further research is required on recovery modalities and whether the development of specific physical qualities can positively influence transient (e.g., within match), acute (e.g., following a match) and chronic (e.g., over a season) fatigue and recovery profiles in netball players.

Studies investigating sleep found significant reductions in total sleep time following a match compared to training and rest days [66], which was consistent with perceived sleep duration and quality [69]. Napping (> 20 min) on the day of performance appears to improve performance [65] and teams who slept longer reportedly achieve higher tournament positions [3]. Given the importance of sleep and disruption following a match, future research should assess the efficacy and effectiveness of sleep hygiene strategies.

### Injury

#### Epidemiology

Of the 45 studies to investigate injury in netball, 31 (67%) had an injury epidemiology focus (Supplementary Table S3). These studies involved a wide range of cohorts including populations such as club [42, 71–76] and state or national level [32–34, 75, 77–80], whilst 23% (*n* = 11) of included studies reported netball injuries relative to “general population” patients [45–47, 81–88]. Studies also examined netball injury in both junior/youth [72, 87, 89] and senior/open age [77–79] athletes. The majority of epidemiological studies collected data over extended periods of a year or longer [32, 45–47, 74, 81–88, 90–93]. However, some studies utilised data representing much shorter periods, e.g., tournament weeks [71–73, 76, 94] or days [33, 34, 42, 75, 77, 78, 80].

Various approaches were taken when defining or classifying injury amongst the included studies. Some based classifications on accepted criteria [73, 78], others utilised broad definitions including, for example, aspects such as an injury that occurs whilst participating in sport, that leads to either a reduction in the amount of level of sports activity, or the need for advice or treatment [30]. Finally, narrow definitions such as “trauma to a specific body part resulting in cessation of play” were also utilised [32].

A common theme of included studies was the reporting of injuries relative to hours of exposure [31, 33, 34, 72, 74, 76, 80, 94] or number of athletes [32, 34, 42, 73, 75, 81, 88, 89, 91–93]. Injury incidence was commonly reported between 12 and 14 injuries per 1000 exposure hours [30, 31, 34, 74, 76]. In addition to injury incidence and prevalence, the activity participants were performing (e.g. matches or training) when the injury occurred was also considered [33, 78], as was the specific movement or mechanism (e.g. landing, contact, overuse) involved [31, 32, 45, 71, 75, 77, 85, 88, 94]. Furthermore, the type and/or site of injury was commonly reported [30, 32, 33, 42, 47, 71, 73–77, 79, 80, 82–84, 86–91] with lower limb injury, particularly to the knee and ankle, common [30, 32, 42, 45, 71, 74, 75, 79, 80, 83, 84, 88, 90, 91, 94]. Whilst a systematic review of the mechanisms of non-contact knee injury has recently been carried out [95], the volume of injury epidemiology research in netball may warrant a systematic review and meta-analysis to provide researchers and practitioners with a high level of evidence regarding incidence and prevalence in addition to injury mechanism and site. Additionally, given there is no current evidence on the epidemiology and burden of concussion in netball, research in this area is also warranted.

#### Risk and Prevention

The remaining 14 injury studies included in the review had an injury risk, influence, or prevention focus (Supplementary Table S4). Junior (*n* = 4, 29%)[89, 96–98] through to open age players (*n* = 8, 57%)[10, 35, 99–104] were investigated and a variety of athlete performance levels were found within the studies including, school or club [96–101, 103, 105], inter-district [99–101], state or elite [102, 104, 106] and international [35]. Although a range of athlete cohorts have been used in netball injury and prevention research, there may be value in extending this work, particularly in higher-level athletes as this group appears underrepresented in current studies.

A common purpose of these studies was the analysis of injury risk factors. These included prior injury [99] and the role of physical capacity (including movement quality) and anthropometry [89, 98, 101–104]. Other studies examined the impact of injury prevention strategies [35, 96, 97] and mechanisms/movement patterns involved in specific injuries (e.g. anterior cruciate ligament (ACL)[106]). Further work analysed the impact of netball on balance and postural sway [105]. Despite the large number of injury epidemiology studies (Supplementary Table S3) there is relatively little work examining injury risk and prevention strategies in netball and these could be the focus of future research.

Given the range of purposes of the studies included in this section, it is not surprising that a variety of data were collected. The most common related to injury history [35, 98, 99, 101–105] whilst balance (e.g. STAR excursion test) was also commonly assessed [99, 101, 105]. Some studies measured physical capacity (e.g. vertical jump) [96, 101, 104]; others reported somatotype and anthropometric variables [98, 102, 103]. In addition, performance on movement screens [98] and mobility measures [89] were utilised along with subjective questionnaires relating to the landing technique [97].

The results of studies assessing injury risk and the value of prevention programs were varied. For example, no difference in balance between participants with and without previous ankle sprain was demonstrated in one study [99] whilst the odds of ankle sprain were four times higher when STAR excursion posterior and medial direction was < 77.5% leg length [101], and a large proportion of knee injuries were associated with knee valgus [106]. Similarly, studies examining the role of somatotype in injury risk produced equivocal results [102, 103].

The findings regarding the role of physical capacity and/or movement skill in injury are also inconsistent. For example, whilst a functional stability program reduced injury occurrence in international level players [35] and a 6-week program improved landing mechanics associated with ACL injury [96], one study found no difference in movement competency, jump performance or ankle dorsiflexion in injured players [98]. In contrast, increased postural sway was evident on the preferred leg in those previously injured [105]. Paradoxically, there is also some suggestion that greater jumping ability and anaerobic fitness were associated with greater injury risk [103]. In comparison to other sports, there is very limited knowledge regarding the role of physical capacity (e.g. strength, aerobic capacity) and other factors (e.g. injury history) in injury risk in netball and this warrants further work. Furthermore, there may be a benefit from studies assessing injury burden (e.g. time lost for specific categories of injury), recurrence rate and the impact of specific rehabilitation protocols.

### Match Characteristics

The details of the 24 studies investigating match-play in netball are displayed in Supplementary Table S5. Of those studies, 63% (*n* = 15) have examined match activity profiles [11, 12, 14, 15, 27, 107–116], whilst seven have focussed on technical and tactical aspects [13, 28, 29, 117–120], and two have examined performance outcomes [121, 122]. A large proportion (*n* = 17, 71%) of the studies examining aspects of match play involved elite-level competition [12, 15, 27–29, 107–112, 117–122] and all (except one study [114]) evaluated female netballers.

#### Activity Profile

A range of metrics have been reported when examining the activity profiles of netball, including speed and distance, and non-locomotor activities (e.g., jumping). These were obtained using various approaches such as notational analysis, computerised analysis systems (e.g. Dartfish) [12], Inertial Measurement Units (IMU; accelerometers, gyroscopes, magnetometers) [113], Global Positioning Systems (GPS) [113] and Local Positional Systems (LPS) [112]. The accelerometery derived metric PlayerLoad™ was used to quantify external load in nine of the activity profile studies [11, 14, 15, 110, 111, 113–116]. The predominance of technologies such as IMU and LPS to quantify the activity profile of netball is likely a function of many studies being conducted using high-level athletes participating in indoor matches, which precludes the use of technologies such as GPS commonly used in outdoor team sports.

A consistent theme across studies was the analysis of positional differences [11, 14, 15, 108, 110, 111, 113, 115, 116], with centre-court players displaying higher external loads than both GK and GS [11, 15, 108, 110, 111, 113, 115, 116]. In addition, a small number of studies has compared the activity profiles of different groups (e.g. male vs female, differing standards) [11, 14, 113, 114]. The ongoing development and increasing availability of measurement technologies provide an opportunity to develop a more detailed understanding of netball activity profiles at various performance levels.

#### Technical-Tactical

The studies examining technical-tactical analysis have quantified a variety of common netball actions such as passing, interceptions, turnovers, and shooting percentage, all including elite level players [28, 29, 117–119]. An interesting finding is that experts appear to execute more passes under low levels of defensive pressure than developmental athletes and this resulted in more successful passes being completed by the elite players [29]; however this was the only study to compare levels of performer. In addition, one study has examined changes in technical-tactical aspects over time [118] and another study investigated potential strategies regarding the new ‘two-point’ rule in Suncorp Super Netball [120]. Further work examining differences in technical-tactical aspects between levels appears warranted and this may assist with both talent identification and training programme design. Furthermore, more research examining links between game events and performance outcomes (e.g. win vs loss) may provide coaches with important information for both training programme design and tactical decision making.

#### Performance

The impact of factors including travel and fixture scheduling on performance (e.g. win *vs* loss, points margin) has been examined in two studies [121, 122]. The small volume of work in this topic limits the ability to definitively determine the importance of these aspects to netball performance and should be a focus of future research.

### Nutrition

Three studies have investigated nutritional outcomes within netball (Supplementary Table S6) [6, 123, 124]. One study investigated the hydration status of international athletes [6], showing sweat and fluid intake rates of approximately 250 and 80 ml·h^−1^, respectively. The other two studies investigated the energy intake, expenditure and appetite of junior athletes at the club [123] and school [124] level, identifying alterations in appetite in response to netball exercise. However, this is yet to be investigated in senior or elite-level netball athletes. Furthermore, investigation into nutritional and dietary behaviors of these athletes may assist in the provision of nutritional interventions.

### Physical Qualities

Thirty-seven studies have investigated the testing methods and physical characteristics of netball athletes (Supplementary Table S7), ranging from junior (*n* = 3) [4, 125, 126] to elite (*n* = 12) [18, 48, 49, 127–135] level. Twenty-two percent (*n* = 8) of studies specifically investigated the validity and/or the reliability of tests or testing outcomes [133–140] and 43% (*n* = 16) had a primary emphasis upon testing physical characteristics [16, 18, 20, 50, 125, 129–132, 141–147], with an additional four studies quantifying anthropometric characteristics [49, 51, 148, 149]. Finally, 24% (*n* = 9) of studies investigated the effects of different training interventions on changes in physical characteristics and performance [4, 48, 126–128, 150–153].

#### Testing

The 505 CoD test has been shown to demonstrate acceptable between-day reliability when 1–2 familiarisation sessions are provided [138]. Furthermore, the 30:15 intermittent fitness test (30:15IFT) may be able to detect changes in high-intensity running performance across a training mesocycle [139]. In comparison, the ‘Net-Test’ [134] and Netball Specific Fitness Test [140], two sport-specific assessments, have demonstrated acceptable reliability and may be able to discriminate between athletes of different playing standards. Similar trends have been shown within the Reactive Agility Test and Planned Agility Tests [135], although it should be noted that the typical variation between-days for these tests are still unknown. Finally, the GRF produced during a single leg horizontal jump can provide reliable outcomes when assessing balance [136], while the Netball Movement Screening Tool is reliable when implemented by practitioners with similar levels of experience with the tool [137]. However, it has not been ascertained whether the Netball Movement Screening Tool is a valid method for detecting increased injury risk for netball players. Considering these findings, the reliability of a narrow scope of tests has been assessed. Thus, further information is still required to ascertain the reliability and validity of a range of different tests and screening methods that assess different physical qualities.

#### Physical Characteristics and Anthropometrics

It has been demonstrated that, when compared to sedentary controls, netball athletes are taller and have greater lean body mass [148, 149]. Additionally, athletes at higher playing standards are taller and demonstrate greater sprint and CoD ability [18, 125, 132, 146], while centre-court players have greater fitness and jumping ability than other positional groups [16, 144]. However, it appears that body fat percentage may not be able to discriminate between playing levels [49, 51]. It has been shown that stronger athletes demonstrate greater acceleration (i.e., 5-10 m), CoD, and vertical jump [145], while the Functional Movement Screen has moderate relationships with trunk stability and CoD ability [130]. Finally, small asymmetries in lower limb stability and vertical hop performance have been shown to occur in club level players [141], while a high occurrence (63%) of general joint hypermobility has been observed in elite players [131]. From these findings, it is clear that differences in athletes are prevalent. However, a lack of systematic physical profiling has been completed (e.g., a standardised testing battery across age groups and playing standards) which is illustrated in a large number of tests, standardisation protocols, and outcome measures reported. Thus, researchers and practitioners should endeavour to work together to develop valid and reliable testing batteries that provide a comprehensive overview of the netball athlete.

#### Training Interventions

Plyometric and strength training can induce favourable adaptations in measures of isometric strength, power, and CoD, while decreasing potential injury risk factors (e.g., peak landing forces) [48, 126, 127, 151]. Improvements in physical characteristics may be greatest in anaerobic qualities, which may be related to previous training exposure [128]. Additionally, training outcomes may be augmented through the use of blood flow restriction or hypoxia when using low relative intensities during resistance training [151]. However, practitioners must be wary of rapid improvements in physical performance during initial training periods and may require systematic alterations in the training stimulus to promote continued performance improvements [127]. When programming, it may be prudent for practitioners to implement specific training interventions. The NetballSmart Dynamic Warm-up has been shown to improve some performance outcomes (i.e., the vertical jump and prone hold) [4], the inclusion of barefoot training and backwards running may enhance CoD ability [150, 152] and a combination of core stability, gluteus medius strengthening and proprioceptive exercises may enhance dynamic postural control [153]. These findings indicate that moderate to large improvements in physical performance occur with training, although further research is still required to elucidate the effects of strength and power training in well-trained netball athletes and whether changes in these physical capacities can reduce injury occurrence.

### Psychology

Thirteen studies have investigated an aspect of psychology in netball (Supplementary Table S8). Five studies (38%) focused on motor learning and decision making [21, 154–157]. Other studies investigated a range of psychological skills or interventions [7, 22, 158–163], including communication [22], anxiety [158], stressors, [159], behaviour [160], team cohesion [161], and imagery [7]. The cohorts investigated ranged from club to elite and international, with six studies (46%)[154–157, 159, 160] investigating international level athletes of a range of ages (U17 to open age). Three studies investigated differences between playing standards or levels [155–157], while three studies made positional comparisons [22, 155, 162]. However, further research is required on the psychological skills of elite netballers and the effectiveness of mental skills training. Given the high prevalence of injury in netball (Supplementary Table S3), research into the psychological effect (i.e., anxiety and stress) of injuries and the impact of psychological support during injury rehabilitation would also be beneficial.

#### Motor Learning and Decision Making

Decision-making was found to be a key discriminator between highly skilled and less skilled performers, evident through greater accuracy in a decision-making task [155–157], while Richards et al. [21] demonstrated the ability of a coach to influence an elite team’s decision making process. Positional comparisons demonstrated limited position specificity in perceptual-cognitive skills in decision-making tasks [155] but GA and WD players have been found to possess greater coping skills compared to other positions [162].

### Training Load

Four studies have investigated the external load of training in netball [5, 164–166] (Supplementary Table S6), with the majority (n = 3) investigating professional level cohorts. Three studies compared the workload of training to competitive match-play [5, 164, 165], two of which investigated specific training dills [5, 164]. One study highlights the need to combine internal and external workloads when monitoring elite netball athletes, suggesting the use of session rating of perceived exertion and CoD as the ideal combination [166]. However, further investigation is required into the training load and training practices of netball across the range of playing standards and age-groups.

## Conclusion

**Fig. 4:**
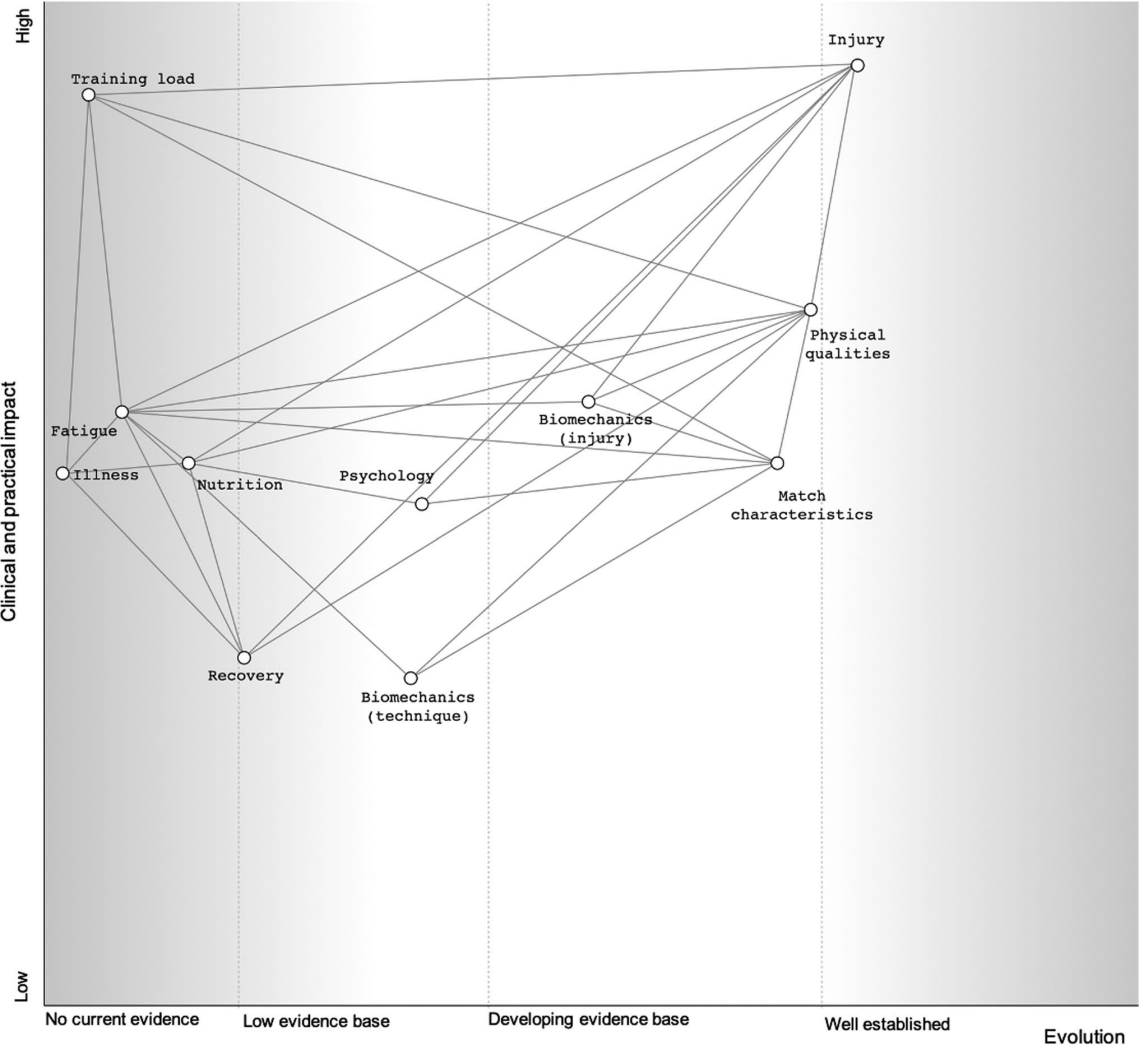
A visual representation of the current evidence base and the clinical and practical impact of netball sport science and medicine topics. Note: Lines between topics highlight potential links and relationships between the areas

This scoping review has identified 150 studies, based on the search criteria used, examining the applied sport science and medicine of netball, with an increase in research seen in recent years. The majority of research originates from Australia in female netball athletes. Across the eight sport science and medicine topics, physical qualities was the first topic studied (1981 [129]), followed by injury (1986 [42]), which has remained a priority of research across the years, likely due to the high injury rates reported in both elite and recreational netball (Supplementary Table S3). It is apparent that topics such as nutrition, training load, and fatigue and recovery are under-investigated within the netball population, likely due to the increased cost of such research due to the technologies required, and perhaps lack of funding compared to other professional sports. Whilst the research has been grouped into eight sports science and medicine topics in this scoping review there are relationships and links between each topic (Fig. 4). This is evident in studies identified in the review overlapping between different topics, which means developing the research in one area has the potential to impact upon another in practice and research. Furthermore, although a systematic search of studies investigating topics within the applied sport science and medicine of netball was carried out, it must be acknowledged that other studies may exist that were not identified by the search terms.

This scoping review summarises the current evidence base and key findings that can be used in practice to enhance the applied sport science and medical support to netball athletes across a range of playing standards, and support the growth of the sport. However, it is apparent the sport of netball is still under-researched.

### Gaps in the Literature and Future Recommendations

Whilst research into the applied sport science and medicine of netball is increasing, this review has identified gaps in the current literature. Areas for future research direction can be guided based on the current level of evidence, alongside the consideration of what is useful in practice [167], demonstrated in Fig. 4. Table 2 summarises the identified gaps and provides some directions for future research. Whilst the process of determining what is useful and impactful (Fig. 4 and Table 2) is subjective, it may prove valuable for both researchers and policy makers in identifying areas to focus their future research.

**Table 2:** Examples of future research directions in topics for which the current evidence base is limited or conflicting, and in topics where there is no current research

Research topic	Focus	Current evidence base		What is the clinical and practical relevance of this research? (i.e., impact on policy and practice)	What is the priority for this research to be carried out? (low/medium/high)
		Limited	Conflicting		
*Current evidence base is limited or conflicting*					
Biomechanics/ injury	Landing technique influence on injury risk	Yes	No	Could direct landing training programmes undertaken at all levels with potential for recommendations and coach training to infiltrate community netball	Medium
Biomechanics/ injury	Lower limb movement screening and injury risk	Yes	Yes	Any links/associations could direct effective screening content	Medium
Injury risk and influence	Role of physical capacity and other factors. Particularly with high-level athletes	Yes	Yes	Would enable a higher standard of decision making and athlete care through implementing training programmes that could mitigate/improve risk	High
Recovery	Effective recovery modalities; specifically during congested fixtures	Yes	No	Current wider research on recovery modalities are extendable to netball. Best strategies during periods of congested fixtures could benefit teams test-series or tournaments	Low
Match-play	Differences in technical-tactical characteristics between levels of play	Yes	No	Would support in directing training progressions through playing careers	Medium
Performance	Influence of fixture scheduling on individual and team performance	Yes	No	Could support in directing set up or scheduling of major events	Medium
Physical qualities	Normative standards and reliability of tests relative to the position and level of the athlete	Yes	No	Would support talent identification and development, and monitoring the progress of a programme	Medium
Physical qualities	Effects of strength and power training in netballers	Yes	No	Could direct more specific training and preparation for performance, but some current wider research could be extended to the sport	Medium
Training and match load	The quantification of external and internal training load in senior professional and international athletes	Yes	No	Could influence daily and seasonal training practices	High
Training load	The appropriateness of training in preparation for match-play	Yes	No	Would allow for more effective training, robust programming, return to play plans and athlete development	High
*No current research*					
Biomechanics/ injury	Ground reaction forces interaction with injury outcomes/risk			Useful to assist in developing mechanisms to mitigate injury risk	Medium
Injury epidemiology	Systematic review/meta-analysis on injury epidemiology			Quick and effective information to inform policies and an opportunity to decrease injury risks at all levels	Medium
Injury epidemiology	Injury, and concussion, burden and recurrence rate at different age groups and playing standards			Important for long term health of player population; would enable the evaluation of current practices and development of programmes to try reduce recurrence	High
Injury risk and influence	Impact of specific rehabilitation protocols			Would inform practice to a greater depth than what already is delivered and could be used to update current practice guidelines	High
Fatigue	The influence of physical qualities on transient, acute and chronic fatigue and recovery profiles			Would impact on training planning, modalities and scheduling to improve tolerance and recovery, especially in congested test series. Understanding the dose response relationship of netball would enhance specificity of training and adaptations	Medium
Recovery	The efficacy and effectiveness of sleep hygiene, with a focus on recovery and performance			Could guide the choice of appropriate recovery strategies but wider research on recovery is extendable.	Low
Match-play	Influence of game events and interactions on performance outcomes and/or activity profile			Could impact on how game is played/approached, and the training undertaken to prepare	Medium
Match-play	Fast Five competition			Could support the preparation for players transitioning between competitions	Low
Nutrition	Energy expenditure in senior and age group elite athletes			Would improve level of support to players, contributing to nutritional intervention. Would link to player health, particularly around RED-s	Medium
Nutrition	Nutritional behaviours of elite athletes, influence of age and experience			Could guide nutritional interventions and help education and management around RED-s.	Medium
Physical qualities	Relationship between physical qualities and match-play activity profile and/or recovery kinetics			Would impact upon training specificity and prescription and inform the direction of many of the other fields	Medium
Psychology	Injury; influence of psychological support during rehabilitation			Would impact on the support provided to players during rehabilitation, especially long term return to play processes, but some wider literature could be extended to netball	Medium
Psychology	Psychological skills of elite athletes, the effect of mental skills training and impact of team dynamics and culture			Would enable more sport specific psychology support	Low
Training load	The quantification of training load in across age-grades and playing standards			Would improve understanding of Long-Term Player development in netball and specifically in female populations	High

Notes on the clinical and practical relevance and research priority for each topic, based on the potential impact, are also provided

## Supplementary Information

Below is the link to the electronic supplementary material.

Supplementary file1 (DOCX 103 kb)

## Declarations

### Availability of data and materials

Data supporting this review are available from the corresponding author upon request.
