# Supplementary material for: The Applied Sports Science and Medicine of Netball: A Systematic Scoping Review
Source: Sports Med. 2021 Jun 4;51(8):1715–31. doi: 10.1007/s40279-021-01461-6 (PMC8310515; doi:10.1007/s40279-021-01461-6)
Supplement: Supplementary file 1 — Supplementary file1 (DOCX 103 kb) [file 40279_2021_1461_MOESM1_ESM.docx]

| Supplementary Table S1. Characteristics, outcome measures and key findings on 'biomechanics’ studies (n = 14) | | | | | |
| --- | --- | --- | --- | --- | --- |
| Study | Cohort/ sample size (n) | Participant characteristics: age, height, body mass | Study purpose | Outcome Measures | Key findings |
|  |  |  |  |  |  |
| Delextrat & Goss-Sampson [61] | Junior & senior county, junior n = 6, senior n = 6 | Junior: 14 ± 1 yrs 174 ± 4 cm 65 ± 8 kg Senior: 19 ± 4 yrs 175 ± 9 cm 72 ± 10 kg | To describe and compare the kinematics of goal shooting in netball in junior and senior players. | Static variables: joint angles of knees, shoulders, elbows and right wrist, trunk lean, distance between heels, position of ball. Dynamic variables: timing between actions, angular velocities, ranges of motion from start to release. | Seniors showed a difference (p<0.05) between the right and left elbow angles at the start of forearm extension and a short delay between the movements involved in the shooting action. The ranges of motion were not significantly different. |
| Hetherington et al. [19] | State level,  n = 1 | 25 yrs | To investigate the efficacy of a combination of tools to describe the kinematic and kinetic mechanisms underlying the netball shoulder pass. | Segmental movements analysed from video and force platform data. Instantaneous angular velocity and acceleration of body segments. | Power developed primarily in the muscles of shoulder, arm and forearm. A large and rapid negative acceleration in the upper limb was found after the propulsion phase. |
| Steele et al. [62b] | State, district and university, n = 10 | 21 ± 1 yrs 170 ± 6 cm 64 ± 8 kg | To examine the influence of different synthetic sport surfaces on ground reaction forces at landing during a simulated netball task. | Landing on 12 different surfaces: bitumen, concrete, sand filed synthetic grass (3 variations), synthetic rubber sport surfaces (7 variations) Kinetic variables: Maximum peak VGRF, initial peak VGRF, peak braking forces. | Changes to the physical properties of synthetic sport surface can influence the impact loads and braking forces experienced with landing in netball. The rubber surfaces demonstrated lower frictional break forces (p<0.05). |
| *‘Injury risk' focused studies* | | | | | |
| Boey & Lee [52] | Elite, n = 21 | 23 ± 4 yrs  178 ± 4 cm  63 ± 5 kg | To investigate if knee moments during weight-acceptance phase of a forward single-leg jump for maximal distance are correlated to reach scores in the Y-balance Test. | Single leg hop: distance and knee moments  Y-balance Test: distance normalised to limb length. | Strong positive correlation between posteromedial reach and externally applied flexor moments (p<0.01, r = 0.56). No correlation between valgus moments and y-balance test reaches. |
| Collings et al. [53] | High-performance and inexperienced, n = 46 | High performance: 18 ± 2 yrs 177 ± 6 cm  67 ± 6 kg Inexperienced: 22 ± 3 yrs 170 ± 5 cm 64 ± 7 Kg | To determine whether young high-performance netball players exhibit different landing mechanics compared to inexperienced controls. | Lower-limb kinematics and kinetics during a CMJ: flexion and range of motion of hip, knee and ankle, peak vertical ground reaction force, knee valgus, knee moment, knee internal rotation angle, knee internal rotation moment. | Netball group landed with less contribution from knee extensors for the non-preferred leg (p<0.001, es = 1.10). No other significant differences but *small* to *moderately*  grater ankle dorsi flexion, knee and hip flexion and range of motion in the netball group.  Experience not enough to develop low risk landing mechanics. |
| Greene et al. [54] | Elite, n = 10 | 18 ± 2 yrs  178 ± 4  70 ± 9 kg | To identify if changes in external ankle support mechanisms effect the range of motion and loading patterns at the ankle and knee joint during a sidestep cutting manoeuvre in netball players. | Kinetic variables: ROM in ankle and knee  Kinematic: ankle and knee moments, VGRF, medial GRF, break GRF, propulsive GRF. | Brace condition reduced ankle join ROM in the sagittal plane compared to the netball shoe but no other significant changes observed. All shoe conditions produced excessive knee valgus moments in the cutting cycle. |
| Fox et al. [55] | Sub-elite, n = 32 | 23 ± 3 yrs 171 ± 8 cm 68 ± 8 kg | To examine the biomechanical relationship between a single-leg squat and a netball-specific leap landing to examine its utility within netball-specific ACL injury risk screening. | Kinematic variables:  - hip and knee: internal/external rotation, abduction/adduction and flexion/extension - ankle: internal/external rotation, inversion/eversion, dorsiflexion/plantarflexion during single leg squat task. Kinetic variables: ground reaction force data and join moments for each joint during the leap landing. | Biomechanical patterns in the lower limb during the single leg squat are associated with a netball specific leap landing task. Hip external rotation, knee flexion, abduction and internal rotation, ankle dorsiflexion and eversion were linked to high risk movement strategised for ACL injury during the landing task. |
| Hopper et al. [8] | Elite, n = 15 | 23 ± 4 yrs 172 ± 7 cm 68 ± 9 kg | To investigate the effects of bracing and taping on muscle activity, rearfoot motion and VGRF during landing similar to that performed in netball. | Single leg jump onto force plate. Kinetic variables: peak VGRF, time to VGRF, EMG data for muscle activity. Kinematic variables: joint angles of rearfoot and Achilles at foot strike. | Lower activity for the gastrocnemius and peroneus longus muscle groups when subjects were braced p<0.0007). Kinetic and kinematic variables were not affected by bracing or taping during landing. |
| Mason-Mackay et al. [56] | School, n = 20 | 16 ± 1 yrs  66 ± 7 kg  172 ± 5 cm | To investigate the impact of lace-up ankle braces on landing biomechanics. | Leg, knee, and ankle stiffness, knee/ankle stiffness ratio, knee and ankle sagittal.  Excursion, peak vertical ground reaction force, time-to-peak vertical ground reaction force, and loading.  rate during drop jump, drop land and netball specific task. | Kinetic changes (*small*) combined with observed *small* increases in ankle stiffness suggests participants able to compensate via changes in joint excursions to prevent increased loading. |
| Otago [57] | *NR,* n = 14 | 22 ± 4 yrs 171 ± 7 cm 66 ± 7 kg | To determine whether changes to the footwork rule would reduce the ground reaction forces on landing. | Peak vertical ground reaction force, peak resultant breaking force, time to peak VGRF, time to peak braking force.  Breaking force and vertical loading rates. During 5 landing conditions (3 legal footwork, 2 illegal footwork). | No significant differences found between each respective legal and extra step techniques. Run on techniques exhibited lower peak forces, longer attenuation times and lower loading rates than the pivot or two good landings.  No advantages gained from taking an extra step. |
| Sinclair et al. [9] | University, n = 20 | 21 ± 2 yrs 167 ± 4 cm 61 ± 7 kg | To investigate the effect of prophylactic knee brace on knee joint kinematics and kinetics during netball specific movements. | Patellofemoral joint kinetics (force and pressure), knee kinematics (range of motion, minimum and maximum angle of sagittal, coronal and transverse plane) during run, cut and jump movements with and without knee brace. Subjective ratings of comfort and stability. | The prophylactic knee brace did not significantly influence joint kinetics during any movement. But the brace reduced internal/external rotation range of motion in all movements (p< 0.05) and helped increase perceived knee stability (p<0.05). |
| Sinclair et al. [58] | University, n = 20 | 20 ± 1 yrs 166 ± 5 cm 52 ± 7 Kg | To investigate the effect of prophylactic knee bracing on knee joint kinematics and kinetics during simulated centre pass single- and double-limb deceleration tasks. | Kinetics: patellofemoral (force stress and load rate), patellar tendon (force and load rate), ACL (force and load rate). Knee joint kinematics: ankle of foot strike and ROM in sagittal, coronal and transvers plane, peak flexion, abduction and external rotation.  Subjective ratings of comfort and stability. | Participants perceived the prophylactic brace to improve joint stability (p<0.001) but the brace did not significantly effect any kinetic/kinematic parameters linked to the aetiology of injury in netball specific deceleration tasks on both single and double limb. |
| Smyth et al. [59] | State, n = 53 | 18 (16 to 23) yrs | To assess whether ankle tape applied by a physiotherapist or self-applied by the athlete results in a change in proprioception and whether it is maintained during a netball session. | Proprioceptive acuity assessed using the AMEDA. | Increase in AMEDA score observed with self-applied tape and physiotherapist applied tape (p<0.05) after initial application. Improvements maintained during a netball session with no significant different post session for both application methods (p>0.05). No significant difference in AMEDA scores between self-applied and physiotherapy applied tape. |
| Stuelcken et al. [10] | State level, n = 10 | 18 ± 2 yrs 178 ± 4 cm 70 ± 9 kg | To determine frontal plane loading patterns in the knee during a single leg landing, investigate player adherence to guidelines on effective and safe landing and assess whether the guidelines are applicable for coaches working with high-performance netball athletes. | Kinetic variables: ground reaction force Kinematic variables: knee, hip and ankle angles Net joint moments. Qualitative assessment of knee and foot alignment. | For most players internal valgus moment was the largest frontal plane knee moment during the landing phase. For 6/10 of the players rapid change to an internal knee valgus moment coincided with hip adduction. |
| Vanwanseele et al. [60] | Elite, n = 25 | 18 ± 2 yrs  179 ± 4 cm  70 ± 8 kg | To investigate the effect of external ankle support on the ankle and the knee joint during a netball specific landing task. | Kinematic variables: ROM, angular velocities  Kinetic variables: peak VGRF, ankle and knee moments. | Ankle brace significantly reduced ankle ROM in frontal plane but with no changes in the knee joint loading. High top shoes increased the peak rotation moment 15%. Brace and high-top increased the peak ankle plantar flexion moment.  Ankle braces can be used to restrict ankle ROM during landing without increasing the load on the knee joint. |
| *NR* = not reported*,* GRF = ground reaction force, VGRF = vertical ground reaction forces, CMJ = counter movement jump, ROM = range of motion, ACL = anterior cruciate ligament, EMG = electromyography | | | | | |

| Supplementary Table S2. Characteristics, purpose, outcome measures and key findings of studies within the discipline of 'Fatigue and Recovery' (n = 9) | | | | | |
| --- | --- | --- | --- | --- | --- |
| Study | Cohort/  Sample size (n) | Participant characteristics: age, height, body mass | Study purpose | Outcome measures | Key findings |
| Birdsey et al. [63] | International, n = 11 | 25 ± 4 yrs  180 ± 10 cm  72 ± 8 Kg | To examine the physiological, neuromuscular and perceptual responses to, and physiological demands of, a 3-day International netball tournament. | Perceptual (scores of perceived well-being), physiological (creatine kinase, cortisol and testosterone), and neuromuscular (CMJ height and peak power output) responses across a three day tournament. Internal (heart rate and sRPE) and external (accelerometery) match load. | Consecutive matches revealed dose-response relationship for well-being and physiological function. Match 2 and 3 perceived well-being, peak power output and testosterone decreased *vs.* baseline (*possible* to *most likely*). Match 2 and 3 creatine kinase and cortisol increased (*likely* to *most likely*) *vs.* baseline. No change in external load across the tournament. *Very likely* Increase in sRPE from match 2 *vs.* match 3. |
| Higgins et al. [70] | State, n = 9 | 23 ± 5 yrs  176 ± 4 cm  68 ± 7 Kg | To examine the effectiveness of compression garments on physiological and performance markers in a netball match specific circuit. | Cross-over design: compression garments *vs.* placebo *vs.* normal attire Physiological measures: blood lactate and heart rate Performance measures: 20m sprint, CMJ flight times, total distance covered, and velocity. | No significant interactive effect of compression garments observed on performance or physiological responses.  Greater distances travelled at > 3 m·s^-1^ were observed in compression garments (ES = 0.86), but no significant difference found. |
| Juliff et al. [64] | Elite U19 and U21, n = 10 | 20 ± 1 yrs  182 ± 5 cm  77 ± 9 Kg | To examine the influence of contrast showers and contrast water therapy on recovery after a netball specific circuit. | Randomised cross-over design: passive *vs.* contrast therapy *vs.* contrast showers. Physiological measures: core and skin temperature, heart rate. Performance measures: repeated agility test Perceptions of effectiveness. | No significant differences in repeated agility between conditions at any time point. No significant difference in core temperature between conditions. Skin temperature lower (p<0.05) immediately post contrast water therapy and contrast shower conditions. Perceived fatigue scores lower (p<0.05) for contrast water therapy *vs.* passive condition. |
| Juliff et al. [3] | Under 21 State, n = 42 | 19 ± 1 yrs  180 ± 6 cm  72 ± 8 Kg | To monitor the sleep patterns of netballers during a multiday national competition, exploring differences in sleep variables between high and low performing teams. | Actigraphy derived: time to bed, time of waking, time in bed, sleep duration, sleep onset latency, sleep efficiency. Subjective recordings of sleep quality, bed time and waketime. | ~66% of all sleep nights recoded less than 8 hours a night Strong associations between teams who had longer sleep durations and higher final tournament placings (*r* = -0.58). Top 2 placed teams slept longer (p<0.001), had greater time in bed (p<0.001) and reported enhanced subjected sleep ratings (p = 0.008) *vs.*  lower 2 teams. Post afternoon games athletes had longer sleep vs post evening games (p<0.05). |
| O'Donnell et al. [66] | Elite, n = 10 | 23 ± 6 yrs  80 ± 9 Kg | To measure psychophysiological stress markers during a competition, an intensity-match training and a rest day and to determine their relationship with sleep indices. | Psychophysiological stress markers: saliva cortisol and perceived stress levels Actigraphy derived: time to bed, time of waking, time in bed, sleep duration, sleep onset latency, sleep efficiency. | Cortisol levels higher (p<0.01) immediately post netball match and at 10pm compared to intensity matched training session and rest day. Total sleep time (p<0.01) and sleep efficiency (p<0.05) lower following a match vs training session and control. Total sleep time post-match = 6:03 ± 1:51 (h:mm). |
| O'Donnell et al. [69] | Elite  Part 1: n = 10  Part 2: n = 11 | Part 1:  23 ± 6 yrs  80 ± 8 kg | To evaluate the perceptual (part 1) and objective (part 2) sleep the night before a game, the night of a game and the night following a game. | Perceptual: sleep duration, sleep quality, time slept. Objective, actigraphy derived: time to bed, time of waking, time in bed, sleep duration, sleep onset latency, sleep efficiency. | Across a competitive season, large sleep impairments demonstrated from competing at night. Both perceptual and objective measures of sleep times reduced on the night of a game compared to the night before (p<0.05). Total sleep time post-match = 6:46 ± 0:47. |
|  |  | Part 2:  23 ± 4 yrs  77 ± 8 Kg |  |  |  |
| O'Donnell et al. [65] | Professional, n = 14 | 23 ± 6 yrs | The effect of pregame napping behaviours on perceived energy levels, neuromuscular performance and ratings of match performance. | Performance measures: CMJ mean velocity, peak velocity and jump height. Perceptual measures: perceived energy level, perceived match performance. Coaches rating of performance. | When athletes had nap times of less than 20 minutes on the day of competition increases in neuromuscular jump performance (peak jump velocity p<0.05, ES =0.34, small) and coaches netball performance rating (p<0.05, ES = 0.85, moderate) were observed. Large standard deviations observed indicates multiple influencing variables. |
| Russell et al. [67] | Elite, n = 25 | 21 ± 3 yrs  180 ± 6 cm  76 ± 7 Kg | Examine changes in mental and physical fatigue pre-to-post matches. | Mental and physical visual analogue scales. KPIs from match-play: score differential, centre pass conversion, percentage turnover conversion, number of turnovers, shooting accuracy percentage. | Subjective, mental and physical fatigue increase during a netball match (p<0.01). Acute changes in mental and physical fatigue were related to minutes played (r = 0.32, p < 0.01).  Mental fatigue was a separate construct to physical fatigue, with no relationship. No significant relationships between mental fatigue and KPIs of competition. |
| Venter [68] | Elite and sub-elite, n = 215 | 22 ± 4 yrs | Examine the perceived importance of various recovery modalities and differences between men/women, sport and level. | 5-point Likert scale of how important rate contribution of each modality to their recovery. | Sleep, fluid replacement and socialise with friends rated as important across all participants. Recovery modalities should be sport specific: netball players rated prayer as more important (p<0.05) than hockey and rugby players.  Active cool down was only rated by netball and soccer amongst most important recovery modalities. |
| *NR* = not reported, CMJ = countermovement jump, sRPE = session rating of perceived exertion, KPIs = key performance indicators, ES = effect size | | | | | |

| Supplementary Table S3. Characteristics and key findings of epidemiology focused 'injury' studies (n = 31) | | | | |
| --- | --- | --- | --- | --- |
| Study | Cohort/ sample size (n) | Time course | Injury definition | Key findings |
|  |  |  |  |  |
| Belcher et al. [93] | Netball New Zealand membership holders | 10 yrs | N/R | 20-24 yrs age group highest injury incidence over 10 years: 77.8 injuries/1000 players (ankle), 71.6 injuries/ 1000 players (knee).  Youngest age group showed highest increase in injury counts between 2008 and 2017 (84% increase, IRR = 2.0 ankle, 113% increase, IRR = 2.5 knee).  Older age groups had higher risk of ankle and knee injury than youngest group (IRR 1.9 to 2.2, p<0.001). |
| Bissell & Lorentzos [73] | Netball: club, n = 37 | 12 weeks | (Oslo Sports Trauma Research Center questionnaire) | Prevalence of overuse injuries = 78% 25% of players reported significant injuries i.e., reduced or stopped participation. Overall prevalence per anatomical area: knee = 47 %, ankle = 47 %, shoulder = 22 % Age, playing experience and use of support influenced injury prevalence, but no effect of training hours. |
| Botha et al. [94] | Netball: junior club or school, n =560 | Tournament | Any physical complaint from the player during a netball match or practice session that forced the player to receive medical attention. | 46 injuries sustained. Injury rate = 22.5 per 1000 playing hours.  91.3% occurred during match-play, 8.7% during practice or warm up session.  56.5% injuries were acute and 43.5% were a reoccurrence. 27% injuries were contact injuries. 30% knee injuries, 28% ankle injuries. 47% ligament injury.  37% injuries occurred in 1st quarter, 37% occurred in 4th quarter. |
| Chan et al. [46] | General population patients, n = 696 | 3.5 yrs | N/A | Netball accounted for 21 (4.3%) of ACL reconstruction patients. |
| Chong & Tan [45] | Female general population, n = 13 | 4 yrs | N/A | Of 13 female ACL cases, 4 were netball. All had mechanism of 'awkward landing'. |
| Coetzee et al. [78] | Netball: U19 U21 and senior national, n = 1280 | 3 tournaments | (Rugby Injury Consensus Group questionnaire) | 192 players sustained 205 injuries, acute injuries = 91%. 95% injuries sustained during match-play 3% in warm up and 2% in practice sessions. 60.8% injuries due to physical contact with another player. Highest injury rate per 1000 hours occurred on cement surfaces (500). |
| Finch & Cassell [81] | General population, n = 1084 | 1 yr | Sport injury = any reported injury occurring during the participation of sport and active recreation. 'Significant' injury = injury that required treatment, interfered with performance of the activities of daily living, and or had adverse effects on participation or performance in subsequent activity. | Netball injury rate = 19 per 10,000 population, 51 per 1000 participants. Netball significant injury rate = 24 per 1000 participants. |
| Finch et al. [82] | General population patients | 5 yrs | N/A | 3.7% of all sports injury emergency department presentations in children were netball related (n = 2165): 54 % upper extremity, 37 % lower extremity, 44 % sprain or strain, 15% bruising, 11% inflammation, swelling, pain, 6% head. 6.6% of all sports injury emergency department presentations in adults were netball related (n = 2587): 64% lower extremity, 28% upper extremity, 58% sprain or strain, 13% fracture, 10% inflammation, swelling pain, 5% head. |
| Finch et al. [74] | Netball: club, n = 379 | 2 yrs | An injury that occurs while participating in sport and leads to one of the following consequences: a reduction in the amount of level of sports activity, the need for advice or treatment and or adverse economic social effects. | Overall incidence rate = 11.3 per 1000 exposure hours (95% CI = 9.8 - 12.9). Number of injuries = 216: 61.2% ligament sprain/tear, 54% ankle, 28% knee, 26% finger/thumb, 25% muscular strain/tear, 17% lower back. |
| Flood & Harrison [83] | General population patients | 4 yrs | N/A | 4596 netball-related hospital admissions. The most common admission due to fractures (30%).  ACL diagnosis account for 17% of admissions (mean age 25.5 ± 7.9 yrs). Rate of admission based on ACL rupture = 0.4 per 1000 participants. Achilles tendon injuries account for 16% admissions (35.8 ± 7.8 yrs). |
| Franettovich Smith et al. [31] | Netball: club, n = 269 | 1 year (season) | All types of damage to the body that occurred as a result of competing, training and/or participating in a physical activity. | 169 injuries sustained by 107 players. Injury rate = 13.8 per 1000 playing hours (32.2 per 1000 players hours during match play, 4.7 per 1000 hours during training).60% injuries lower limb.  Collision with another player most commonly reported mechanics. 27% due to awkward landing. |
| Gwynne-Jones et al. [84] | General population patients | 8.5 yrs | N/A | 24% of 363 acute Achilles tendon ruptures on emergency department database occurred whilst participating in netball. Netball most common cause of injury. |
| Hassan & Dorani [85] | General population patients | 1 yr | Sport injury = a sudden external occurrence while participating in a sporting activity, leading to a personal injury that resulted in presentation to A&E. | 13 out of 255 fractures were netball related, all occurring in the school grounds 3 from a 'fall', 8 from 'struck by ball', 1 from 'collision', 1 from 'other'. |
| Hon & Kock [47] | General population patients | 1 yr | N/A | 3 out of 113 hospital presentation sports related fractures were netball related. |
| Hopper [42] | Netball: Junior (A1 to D4 grades) and senior (A1 to D6 grades) club, n = 3108 | 14 weeks | a) the injury presented required immediate care treatment, b) the area injured presented with some degree of disability. | Total injuries recorded = 158 (5.2 % of population). Highest frequency of injury at higher level of competition (senior grade A = 35 %, junior grade A = 47%). 58% injuries occurred at the ankle, 15% at the knee, 13% the hand, 13% other. Significant association with type of injury and quarter of occurrence - new injuries more frequently occurred during the first quarter, chronic injuries more frequently occurred in the 2nd quarter. |
| Hopper & Elliot [77] | Netball: State open age, U21 and U16, n = 240 | Tournament | A disability that caused pain and some degree of dysfunction. | 32% players reported having an injury at the commencement of the tournament, 23% players sustained a lower limb or back injury during the tournament. Most injuries were ligamentous (41%), with some overuse tendinitis (12%) and 2 players diagnosed with ACL.  29% injuries from incorrect landing, 29% from contact with player, 12% % from sudden stop. Association between lower limb and back injuries with level of competition (p<0.05); open age reported more injuries than younger players. |
| Hopper et al. [71] | Netball: Club (A1 to D6 grades), n = 11228 | 14 week tournament, every 5 years | a) the injury presented required immediate care treatment, b) the area injured presented with some degree of disability. | 608 players presented with an injury (5.4 %), ranged from 3.3% Grade D level to 8.5 % grade A level. Probability of risk = 0.054 per person per match. 84% injuries ankle region (67% lateral ligament complex strains), 8 % knee region (2% ACL, 3% meniscus), 3% hand, 5% other.  >38% players considered incorrect landing mechanism for ankle and knee injuries. |
| Hopper et al. [79] | Netball: State open, U21, U16, n = 204 | Tournament | N/R | 90% of players experienced at least one lower injury during netball career, 53% of injured players had ankle injuries, 19% of all injuries were at the knee joint, 36% shin soreness and 12% retro patellar pain. 23% of reported injured players had a normal foot type, 57% of injured players had pronating foot types with rearfoot abnormalities, and 6% had pronating foot types with forefoot abnormalities. Non-pronating feet occurred in 5% of injured players. |
| Hume & Marshall [92] | General population patients | 10 yrs | Sport injury = an injury occurring in a place of recreation and sport, involving either an organised sporting activity or training. | 139 netball sport injuries, 3.2% of all hospitalised injuries due to sport. Netball injury incidence rate = 89.34 per 100,000 participants in 1988. |
| Hume & Steele [34] | State, n = 940 | 3-day tournament | N/R | Injury incidence rate: 13 injuries per 1000 players, 24 injuries per 1000 playing hours. 131 players incurred injuries. 65% injuries in U17 category. Ankle and knee most frequently injured (14% ankle, 14% knee). |
| Janssen et al. [86] | General population patients | 5 yrs | N/A | Netball activity classified annual ACL reconstructions = 1085. Annual ACL reconstruction incidence per 100,000 participants in netball total (95% CI) = 188 (184 - 194). |
| Joseph et al. [90] | Netball: players registered with Netball Australia, n = 413800 | 12 months | A bodily injury resulting from accident, which is not an illness and which is not of a repetitive nature and which occurs during the period of insurance, and within 12 months of injury, results solely and independently of any other cause in events covered under this policy, and includes any condition resulting from exposure to the elements as a result of injury. | 1239 successful insurance injury claims. Injury incidence rate: 2.936 injuries per 1000 participants. 42% knee, 29% ankle, 57% sprains/ligament damage, 15% fractures. |
| Kirkwood et al. [87] | General population patients; children (0 - 19 yrs) | 2 yrs | N/A | Netball accounted for 8 % (154) of female sport associated injuries presented at two emergency departments.  Largest number of netball injuries occurred at age 14 yrs, 29% injuries to upper limb and 23 % to lower limb. 8% of netball injuries were fractures. |
| Langeveld et al. [33] | Netball: university and national, n = 1280 | 4-6 day tournament | Any physical complaint that a player sustained during a netball match or netball training that forced the player to received medical attention. | Injury incidence rate: 500.7 injuries per 1000 playing hours.  91% injuries = acute, 8.8% = recurrent or chronic. 36% injuries at the ankle, 19% knee joint, 16% wrist, hand and fingers, 12% lower leg/Achilles. Ankle resulted in the majority of serious injuries (42%) 95% sustained during tournament match-play, 3% during warm up, 2 % during practice session. In 61% of cases contact was involved in injury mechanism. Injury incidence highest in 3rd quarter. |
| Otago & Peake [91] | Netball: players registered with Netball Victoria, n = 87331 | 1 year | N/R | Injury incidence rate = 9.49 per 1000 players. 829 injury claims: 85% to lower limb, 9% to upper limb, 3% to spire/torso, 3% to head. Ankle sprain = 31%, knee ligament sprain = 20.5%, knee reconstruction = 13.6%. |
| Pillay & Frantz [75] | Netball: Club, provincial and national, n = 254 | Tournament | Any physical complaint sustained by a player that occurred during a match or training, irrespective of the need for medical attention or time loss from practices or matches. | 62% of players reported an injury: 56% at club level, 59 % provincial players, 84% national players. 38% injuries at the ankle, 29% knee, 7%lower leg/calf, 7% = hand/wrist. Most common mechanism = landing (knee = 19%, ankle = 29%), tripping (knee = 8%, ankle = 6%). Of the injured players, 1.9 injuries per player reported for the past season. |
| Pringle et al. [72] | Netball: youth, n = 1512 | 4 week | Minor injury = one where the players was still in discomfort immediately after the game but able to play the following week Moderate injury = one that presented the plyer from participating in the following weeks. | Injury incidence rate: 13 injuries per 1000 playing hours. 8 minor injuries, 7 moderate injuries. |
| Singh et al. [32] | Netball: International senior, U21 and U16, n = 59 | 5 yrs | Trauma to a specific body part resulting in cessation of play. | 29% players suffered from an injury, 24% players had recurring injuries (all knee or ankle). Knees and ankles accounted for 98% of injuries (knee = 42%, ankle = 56%), with 8 knee surgeries (4 ACL) over the period observed. 53% players attribute injury to poor landing technique, 28% to collisions, 18% poor playing surface, 23% repetitive movements (out of the 40 players that reported reasons). |
| Smartt & Chalmers [88] | General population, n = 56144 | 5 yrs | N/A | 1126 (3%) of public hospital inpatients were netball related; 81% of which female, 19% male. Netball injury incident rate: 123 per 100,000 participants. Participant injury rate increased with age: 325 per 100,000 for 35 - 49 yr olds. 61% of cases were lower leg/knee, 13% forearm/elbow, 6% ankle/foot. Most common diagnoses to lower leg/knee = ACL rupture (22 cases).  Overexertion and strenuous or repetitive movements = most common mechanisms (46%), fall second most common (28%). |
| Smyth et al. [80] | Netball: High performance U19 and U17, n = 192 | 6-day tournament (+4 weeks following) | (Concurrent Injury Definitions Concept Framework) Clinical examination injuries = any health complaint that required the team physiotherapist to assess, irrespective of time-loss. Sport incapacity injury = an injury that required an athlete to miss any duration of math-play during the tournament or where the athlete reported a reduction in capacity during the tournament. | 103 injuries sustained by 80 athletes.  Incidence rate: 89.4 per 1000 playing hours Sports incapacity incident rate: 19.1 per 1000 player hours Lateral ankle ligament sprain = most common injury (14%), foot blisters (11%) and lumbar pain (10%) across both age groups.  Ankle sprains (n = 4), ACL ruptures (n = 3) and concussion (n =3) were the highest reported sports incapacity injuries. |
| Stevenson et al. [76] | Netball: club, n = 379 | 5 month | An injury that occurs while participating in sport and leads to one of the following consequences: a reduction in the amount of level of sports activity, the need for advice or treatment and or adverse economic social effects. | Injury incidence rate: 12.1 per 1000 hours of sports participation. Highest incidence rate in first four weeks of the season = 28.9 / 1000 hours (95% CI: 20.7 to 37.0). 112 netballers sustained injuries (16%). 37% of injuries were ligament strains or tears. |
| N/A = not applicable, N/R = not reported, ACL = anterior cruciate ligament, IRR = Incident rate ratio | | | | |

| Supplementary Table S4. Characteristics and key findings of risk, influence and prevention focused 'injury' studies (n = 14) | | | | | |
| --- | --- | --- | --- | --- | --- |
| Study | Cohort/ sample size (n) | Participant characteristics: age, height, body mass | Purpose | Outcome measures | Key findings |
|  |  |  |  |  |  |
| Attenborough et al. [99] | Club and inter-district, n = 96 | 21 ± 6 yrs 170 ± 7 cm 70 ± 14 Kg | To determine the impact of prior ankle sprain history on the balance of netball players. | Injury: self-reported ankle sprain history. Balance: Star excursion balance test, demi-pointe balance test. | 72% participants reported previous ankle sprain. No differences in any balance measures between participants with or without previous ankle sprain. No difference in balance ability between playing levels. |
| Attenborough et al. [100] | Club and inter-district, n = 96 | Club: 24 ± 8 yrs, 168 ± 5 cm, 69 ± 16 kg Inter-district: 19 ± 4 yrs, 173 ± 7 cm, 72 ± 13 kg | To report the presence of chronic ankle instability within a cohort of netball players and determine any differences in the presence of chronic ankle instability between levels. | Injury: self-reported ankle sprain history Cumberland Ankle Instability Tool - youth, mechanical ankle instability: inversion and eversion. | 69 players previous ankle sprain, 64 (47% of cohort) of which had recurrently sprained ankle.  There was no difference in sprain history between levels (p=0.06). 64% of players with previously sprained ankle had a moderate-severe degree of perceived ankle instability. Previous sprain history did not affect inversion-eversion angles. |
| Attenborough et al. [101] | Club n = 42, inter-district, n = 54 | 22 ± 6 yrs 170 ± 7 cm 70 ± 14 kg | To determine whether pre-season measures of physical attributes and sport specific functional tasks could be identified as risk factors for ankle sprains sustained during netball participation. | Physical attribute measures: vertical jump height, ankle joint laxity, perceived ankle instability, previous sprains, static and dynamic balance. Injury: exposure time, sprains recorded and diagnosed physio/doctor or self-diagnosed. | Injury incidence = 1.74 ankle sprains/1000 hrs of netball exposure. 6.75 ankle sprains per 1000 hrs of netball match-play. 0.40 ankle sprains per 1000 hrs of netball training.  Odds of sustaining an ankle sprain during netball 4.04 (95% CI 1.00 to 16.35) times greater for players who recorded a preseason reach in the posterior-medial direction of star execusion balance test ≤ 77.5% leg length (p =0.02). |
| Elphinston & Hardman [35] | International, n = 17 | 26 ± 3 yrs  172 ± 8 cm 75 ± 15 Kg | To report the implementation of a multidisciplinary approach to injury reduction and performance development in an international netball squad. | Injuries: previous injuries self-reported via questionnaire, head physiotherapist assessed acute injuries during the implementation of the functional stability programme. | Pre functional stability programmes (2001) squad injuries = 22.  Following the implementation (2002), squad injuries = 4. |
| Hopper [102] | Elite (state): open, U21, U16, n = 213 | open age: 24 ± 4 yrs, 174 ± 6 cm, 66 ± 8 kg U21: 19 ± 2 yrs, 173 ± 6 cm, 67 ± 7 Kg U16: 15 ± 0.4 yrs, 169 ± 6 cm, 59 ± 6 Kg | To investigate the relationship between somatype, performance characteristics and the incidence of injury at the Australian Netball Championships. | Injuries: lower limb and back injuries recorded by team physiotherapists. Somatotype ratings of endomorphy, mesomorphy and ectomorphy. | 52 players presented with lower limb or back in jury (24%). No effects of somatotype on the incidence on injury (p>0.05). |
| Hopper et al. [103] | Club: A1 to D6 grades, n = 72 | 21 ± 4 yrs 65 ± 8 kg 171 ± 6 cm | To determine which kinathropometric and performance variables predict injuries in female netball players. | Injuries: lower extremity and back injuries recorded by physiotherapist. Kinanthropometric measurements: somatotype Performance measures: hypermobility, static balance, vertical jump, anaerobic fitness. | 22 injuries recorded in 14 weeks, 59% affecting the ankle region, 18% knee ligaments, 18% back and 5% Achilles tendon. Injuries more common in A1 grade players vs other grades (p<0.001). Injured players had greater jump height, anaerobic fitness and lower endomorphy somatype vs non injured players (p<0.05). After allowing for differences in jumping ability and endomorphy there was no difference between A1 and non-A1 players in risk of injury. |
| Hopper et al. [96] | Youth, n = 23 | 12 ± 1 yrs 163 ± 8 cm 52 ± 9 Kg | To examine the effects of a neuromuscular training programme on lower-extremity biomechanics associated with knee injury during landing, in youth female netball athletes. | Lower limb kinematics and vertical ground reaction force measure in drop jump and double leg broad jump with single leg landing. | A 6-week neuro-muscular training programme can enhance landing biomechanics associated with ACL injury in 11-13 yr old female netball athletes Knee internal rotation angle during the unilateral landing significantly reduced (p<0.05, g >1.00), with large decreases in peak VGRF in both landing tasks (p<0.05). Control group did no demonstrate any significant pre-post changes in response to 6 week study period. |
| McManus et al. [30] | Club, n = 368 | 66% between 16 to 30 yrs | To identify risk and protective factors for injury in non-elite netball. | Injuries: nature of injury, severity and treatment obtained  Baseline questionnaire and monthly phone interviews.  Number of training sessions and or matches played in preceding 4 weeks. | Injury incidence rate: 14 injuries per 1000 playing hours, 66% injuries to lower limb: ankle (32%), knee (17%).  Not warming up pre game = increased risk (IRR [95%CI] 1.11 [1.00 to 1.23], p = 0.048). Not being open to new ideas = increased risk (IRR 1.04 [1.00 to 1.07], p = 0.010). Training at least 4h/week = decreased risk *vs.* < 4hr week (IRR 0.55 [95% CI 0.45 to 0.98], p = 0.039). Not being injured in last 12 months = decreased risk (IRR 0.58 [0.43 - 0.79], p = 0.001). |
| Rodriguez et al. [104] | Elite (professional) n = 10, sub-elite (state) n = 19 | 24 ± 3 yrs 177 ± 7 cm 72 ± 6 kg | To explore the relationship between lower body stiffness and incidence of non-contact injuries in elite and sub-elite netballers. | Injuries: recorded physiotherapist (elite), self-reported (sub-elite) Stiffness: vertical hop test (active vertical stiffness), myometry (quasi-static stiffness). | Lower body injury incidence rate: 11.29 per 1000 exposure hours. 33% calf, 25% ankle, 17% knee.  Elite injury incidence = 19.35 per 1000 hours, sub-elite = 7.13 per 1000 hours No differences in vertical stiffness between injured and non-injured elite players. Injured elite players had greater quasi-static stiffness in the soleus and Achilles *vs.* non-injured players (p<0.05). |
| Smith et al. [89] | Junior, n = 200 | 11± 2.5 yrs | To evaluate the incidence of hypermobility in young female netball players and determine the relation between hypermobility, previous injuries and the use of protective equipment. | Injury: reporting of any injury as a result of netball.  Hypermobility score (Beighton scores). | 35% of players sustained some form of injury whilst playing netball. Most common injuries playing netball: ankle = 42%, knee = 27% finger = 15%. The highest proportion of injuries came from the distinctly hypermobile group (score 5 -9) (p<0.025), 43% reporting injuries, vs non-hypermobile( 21%) and moderately hypermobile (37%). Hypermobility = independent risk factor for netball injury. Beighton scores 3 -4 were 3.4 times more likely to be injured and players with scores of 5-9 were 3 times more likely to have been injured vs non-hypermobile players. For every year netball player, 1.5 times increase in netball injury rate. |
| Stuelcken et al. [106] | Elite (professional and international U21), n =16 | N/R | To describe the game situation, the movement patterns involved and the player behaviour and a potential injury mechanism of ACL injury cases in elite-level netball games. | Systematic video analysis of 16 ACL injury instances: game situation, movement patterns involved, | 13 instances occurred during landing from a jump; split land n = 6, leap landing n = 5, hop landing n = 1, single leg landing n = 1 50% = non-contact, 50% = indirect contact. Indirect contact cases involved some perturbation either in the lead up or at the time of injury.  Two common scenario identified in which a large proportion of injuries occurred (scenario A and B). Knee valgus on collapse occurred in 3 out of 6 scenario A cases and 5 our of 6 scenario B cases. |
| Waterman et al. [105] | Club, n = 27 | 20 ± 4 yrs, 64 ± 8 Kg 173 ± 5 cm | To assess whether participation in a netball games affected the balance parameters of postural sway and the forward lunge, and investigate possible associations between changes in these parameters ad time since injury, perceived injury recovery, grade and position played. | Previous injuries: self-reported via questionnaire. Balance assessment: unipedal stance with eyes closed, and forward lung (postural sway, distance lunged, impact force, contact time). | 52 past injuries reported. Ankle injuries = 55.8%, knee injuries = 19.2 %,shun and calf = 13.5%, foot and ankle = 11.5 %. Increase in postural sway on preferred leg (26.43 ± 42.35, p = 0.005). Decrease in distance lunges on both preferred and non-preferred legs (-4.68 to - 4.80, p = 0.00). No clear pattern emerged with injuries, time since injury and perceived recovery with pathological sway during single leg stance. No significant differences in measures between ankle and knee injury groups. |
| Whatman & Reid [98] | School, n = 166 | 16 ± 1 yrs 172 ± 7 cm 69 ± 10 Kg | To investigate the movement quality, physical performance and prevalence of overuse injuries in secondary school netballers. | Previous injuries: Oslo Sports Trauma Research Centre overuse Injury Questionnaire. Anthropometrics: stature and body mass MC: netball movement screening tool, drop jump, dorsiflexion range of motion. | Prevalence of knee injuries = 31%, substantial knee problems = 10%. Prevalence of ankle injuries = 51%, substantial ankle problems = 24%. No differences in MC score, jump performance of dorsiflexion ROM between players with or without history of knee or ankle problems. Players with history of ankle problems had higher frontal plane knee angle on left side only on the single leg squat *vs.* no ankle problems (p = 0.02, ES = 0.49). |
| White et al. [97] | Junior club, n =287 | N/R | To understand behavioural factors associated with junior community netball players intentions to learn correct landing technique during coach-led sessions as a means of reducing the risk of lower limb injury | Questionnaire assessing players attitude, subjective norms, perceived behavioural control and intentions around the safety behaviour of learning correct landing technique. | Players had positive intentions and attitudes towards learning safe landing technique. Players perceived positive social pressure from significant others.  Players attitudes (p<0.001), and subjective norms (p<0.001) but not perceived behavioural control were associated with strong intentions to learn correct landing technique. |
| ACL = anterior cruciate ligament, VGRF = vertical ground reaction force, ROM = range of motion, MC = movement competency, N/R = not reported | | | | | |

| Supplementary Table S5. Characteristics, outcome measures and key findings on 'match-play' studies (n = 24) | | | | | | |
| --- | --- | --- | --- | --- | --- | --- |
| Study | Cohort/ sample size (n) | Participant characteristics: age, height, body mass | Study purpose | Number of matches (n) | Outcome Measures | Key findings |
|  |  |  |  |  |  |  |
| *‘Activity profile’ focused studies* | | | | | | |
| Bailey et al. [111] | Elite (professional), n = 12 | 26 ± 5 yrs 183 ± 9 cm | To quantify the player load associated with typical player activities in elite netball. | 2 | Accelerometer (x8m-3mini) derived PL.  Video recording coded for locomotor (standing, walking, jogging, shuffling, running) and non-locomotor (goal, pass, catch, jump, rebound, guard, off-ball guard, defend) activities. | Off-ball guarding greatest amount of PL per minute per instance (2.8 ± 1.1 AU·min^-1^), but when expressed per match jogging highest (376.79 ± 239.10 AU·min^-^).  Positional differences in the contributions from locomotor and non-locomotor activities toward total match load.  C greatest overall PL for jogging (p<0.05), WA and WD greatest overall for running (p<0.05), GS and WA greatest overall for shuffling (p<0.05). |
| Brooks et al. [15] | Elite (professional), n = 12 | 26 ± 5 yrs 179 ± 9 cm 71 ± 7 kg | To quantify and describe the position specific physical movement demands of elite level netball match-play | 6 | LPS and accelerometer (Catapult T6) derived: total distance, average speed, velocity zones, PL, PL/min, PL2D, Plslow, PL in each vector, PL bands, total jumps, acceleration density, acceleration density index, total acceleration load, acceleration bands. | Positional differences in match profiles. C covered the greatest total distance (5462.1 ± 169.4 m), total acceleration load (3191.4 ± 115.2 m·s^-1^), total PL (744.4 ± 59.4 AU) and PL2D (464.4 ± 36.8 AU). GD greatest PLslow (382.1 ± 25.7 AU) GS lowest total distance, acceleration load, total Pl and PL2D, but greatest total jumps (82.7 ± 6.8) and acceleration density index (7.21 ± 0.88 m·s^-2^). |
| Cormack et al. [11] | State level club (high standard), n = 17 Recreational club (lower standard), n = 15 | 23 ± 4 yrs 174 ± 7 cm 68 ± 8 kg | To assess the usefulness of load/min as an activity-profile in netball and quantify position-specific profiles. | 5 | Accelerometer (MinimaxX) derived: PL per minute and PL in each axes (vertical, side, forward) per minute, 5 contribution of individual vectors. | Difference in player load between standards. Whole match load/min: higher standard = 10.0 ± 2.5 AU/min *vs.* lower standard = 7.00 ± 1.8 AU/min, 100% *likely lower.*  Centres at both standards *likely* higher load/min *vs.* shooters (~16 22% higher). At the lower standard centres *likely* higher (14.8 ± 26.8%) *vs.* defenders but *unclear* at higher standard. Difference between 1st and 2nd halves *unclear* for higher standard, but lower standard centres saw a *likely* decrease in load/min (-7.7 ± 10.8%). Higher standard accumulated a greater proportion of total load in the vertical plane (*likely*). |
| Davidson et al. [108] | Elite, n = 6 | N/R | To quantify the movement patterns demonstrated by netball players of different positions. | 3 | Notational analysis of movement patterns: standing, walking, jogging, running, sprinting and shuffling. Predicted distances covered. | Change in activity highlighted every 4.1 seconds.  Predicted total distance ranged from 7984 ± 767 m for C to 4210 ± 477 m for GS. C greater than GK and GS (p<0.01). |
| Fox et al. [16] | International, *NR* | N/R | To examine the current game demands of elite level netball with regard to the activities performance and work/rest profiles. | 3 | Computerised analysis system (Dartfish, Australia) derived player movement patterns: Walking, jogging, shuffling, running and sprinting. Game related activities: goal, pass, jump, rebound, guard, off-ball and defend. | Mean duration of work periods = ~2.9 to 6.0 seconds Mean frequency of all work periods = ~ 215 to 259  'Shuffling' most frequent movement across all positions. |
| Fox et al., [109] | International | N/R | To quantify the in-game landings performed by netball players across court positions, with regard to landing techniques and the game events surround the landings. | 3 | Computerised analysis system (Dartfish, Australia) derived landing classifications (double leg landing, split land, side land, leap land, hop land and single leg land) and movement (stationary, walking, jogging, shuffling, running and sprinting) and activities (goal, pass, catch, jump, rebound, guard, off-ball guard, defend) surrounding the landing. | Number of landings: ~152 (GS) to 220 (WA). Differences in distribution of landing techniques across positions. Running and sprinting commonly preceded leap and hop landings (32 to 44%). Leap (92.3%), hop (97.7%) and side (95.5%) landings resulted mostly from a pass. Double landings most frequently employed when attempted a rebound (56.7%). Split land most frequently performed post shot attempt (68.2%). |
| Fox et al. [27] | International, *NR* | N/R | To identify frequently used agility techniques, which playing positions performed manoeuvres more frequently, and how different techniques altered the outcome of evading defensive opponents. | 3 | Computerised analysis system (Dartfish, Australia) derived offensive agility manoeuvres. 5 classification of type of manoeuvres: side-step, shuffle, split-step, stop-and-back, spin. 4 performance classifications: successful and possession, successful and draws penalty, successful and no possession, unsuccessful. | Mid court and attacking positions perform a greater frequency of manoeuvres than defensive playing positions. No effect of manoeuvre type on performance (p>0.05). |
| Graham et al. [144] | State level (championship, division 1, Under 19), n = 28 | 19 ± 5 yrs | To investigate the peak PL per minute intensity across multiple time epochs between positional groups. | 59 (20 Championship, 20 division 1, 19 U19) | Accelerometer (Optimeye S5) derived: duration specific (30 seconds, 1 to 10 minutes) peak player load. Intercept and slope values from power law relationship. | No substantial differences between competitive level. No positional differences at 30 seconds.  1/3rd group substantially lower *vs.* 2/3rd group at 1 to 10 minutes (ES = 0.36 to 1.17) and 3/3rd group (ES = 0.83 to 2.59).  3/3rds group substantially higher than 2/3rds group 2 to 10 minutes (ES = 0.44 to 0.81). |
| Hopper et al. [107] | International, n = 16 (two teams) | N/R | To identify the typical landing pattern and type of passing technique used, the association between landing patterns and passing technique, and the three-way relationship between passing technique, landing pattern and playing position. | 1 | Each pass analysed by observing: 1) the position of the player receiving the pass 2) the team of that player 3) variables related to passing and receiving the ball: type of pass, direction of movement, height of catch, receipt and disposal 4) variables relating to landing patterns: footfall pattern, movement pattern towards the ball, side of landing. | Forefoot landing most preferred landing pattern (57.3%). Most approaches involved horizontal elevation (hop and leap = 24.0%). 25% passes received with foot planted. 24.7% caught the ball following a jump. Low elevation movements (i.e., skip and run) observed 15.8% of the time. Positional differences in type of pass (p = 0.000), height of catch (p 0.013), hand throw (p = 0.000), landing side (p =0.000), landing movement pattern (p=0.000), landing fall = (p = 0.000). Regardless of position, significant association between 1) direction of movement and all landing variables,2) type of pass and all landing variables except footside, 3) height of catch and all landing variables except footside. |
| King et al. [113] | Club, n = 11 Under 19 regional, n = 11 Over 19 regional, n = 12 | Club: 26 ± 4 yrs U19: 17 ± 1 yrs O19: 24 ± 2 yrs | To quantify the movement demands and physiological responses of positional groups and between levels of competition. | 20 (10 club, 6 U19, 4 O19) | Global Positioning System derived (Catapult Optimeye S5): match time, total distance and maximum velocity Accelerometer derived: PL, PL2D, PL in each axes. Mean and peak heart rate. | Differences between levels and positions. O19 representative greater total distance than U19 (p<0.01, ES = 0.33), greater PL and PL in each axes *vs.* domestic (p<0.01, ES = 0.24 to 0.42). C greatest distance covered (4951 ± 3421 m). PL/min range: 8.6 ± 5.7 (GK) to 15.3 ± 10.6 (C). |
| Shaw et al. [115] | School, n = 44 | ~59 to 76 kg  ~162 to 178 cm | To investigate the physical characteristics and physiological demands of match-play and positional differences. | 16 | Global Positioning System derived (Catapult Optimeye X4): total distance, velocity and velocity zones.  Accelerometer derived: PL. | Differences between positions. GS and GK covered less distance (p<0.001) compared to all other positions, with lower maximum velocities (p<0.001) and lower PL (p<0.001). C covered more distance, with highest distance in all speed zones and player load than all other positions (p<0.001). |
| Sweeting et al. [112] | International, n = 12 | 25 ± 3 yrs 180 ± 7 cm | To develop a methodology to uncover the movement sequences performed by court-based team-sport athletes. | 4 | Spatiotemporal data (via Wireless Ad Hock System for Positioning [WASP]) derived velocity and discrete movement patterns/sequences. | 10 frequently reoccurring movement sequences across all positions were discovered. The most prevalent movement features were walking with straight movement and neutral acceleration.  The GD, GA and WA are the most closely related playing positions. Largest dissimilarity between GS and GD (Minkowki distance = 19.64), followed by GS and C (=19.20). The GS is highly dissimilar to all other positions. |
| Tissera et al. [114] | School  Males n = 34, females n = 45 | Males: 15 ± 1 yrs Females: 15 ± 1 yrs | To investigate whether adolescent males and females playing netball differed. | 42 (18 mixed sex, 12 female, 12 male) | Accelerometer (MinimaxX S4) derived: PL, PL per minute, PL2D, Player load in each axes. Video footage coded for netballers specific events: successful catches, total catches, successful passes, total passes, success pass, total violations, total actions, successful actions Efficiency indices. | Overall no differences found between males and females match-play performance (p> 0.05, es = 0.01 to 0.18). Notational skill based comparisons showed male skills were better in mixed than single sex matches. Total PL per min: male = 9.2 ± 2.3, female = 9.2 ± 2.6 AU·min^-1^ For females, 2 physical tests (jump height and YoYo distance) associated with physical match play characteristics (r = 0.45 to 0.64, p<0.01). |
| Van Gogh et al. [116] | Club, n = 11 | 15 ± 1 yrs  175 ± 5 cm  68 ± 6 kg | To examine the activity profiles and physiological demands of youth netball. | 8 | Global Positioning System (SP Pro) derived: distance, velocity, velocity zones.  Accelerometer derived: BodyLoad and impacts  Heart rate zones.  Video coded for skill events: pass/catch, jump/land, shot, rebound, deflection, intercept. | Differences between positions. GS and GK covered less distance (p<0.001) compared to all other positions, with lower maximum velocities (p<0.001) and lower BodyLoad (p<0.001). C covered more distance and BodyLoad than all other positions (p<0.001). |
| Young et al. [110] | Elite (professional), n = 12 | 26 ± 5 yrs, 183 ± 9 cm 73 ± 8 kg | To use accelerometery to quantify plyer load in elite netballers across an entire season. | 16 | Accelerometer (X8M-3mini) derived: PL. | Positional differences in PL per min (p<0.05, except GD, GK and GS). Range = 3.25 ± 0.33 AU·min^-1^ (C) to 1.88 ± 0.18 (GD) AU·min^-1^. Greatest proportion of match time in lowest intensity zone for all positions.  Players optimally grouped into, 1: C, WD, WA, GA and 2: GS, GD, GK, based on playing intensity. Playing intensity higher during matches *vs.* training (1.52 AU·min^-1^ *vs.* 2.60 AU·min^-1^, p<0.001). |
| *Technical-tactical characteristics focused studies* | | | | | | |
| Browne et al. [119] | Elite, n = 67 (7 teams) | N/R | To combine motif analyses and association rules to explore passing patterns in netball. | 8 (+ 3 for validation) | Passing-sequence observations via observational coding (Sportscode). | Most frequent passing style form centre pass: C - GA - WA - GS. The ball was rarely passed back to the player it was received from.  The most confident rule flowed down the right hand side of the court. But 7 out of 10 of the most confident rules demonstrated preference for ball down left hand side. |
| Bruce et al. [29] | International (expert), State (developmental) | Expert: 26 ± 5 yrs Developmental: 17 ± 1 yrs | To investigate the decisions made by exert and highly skilled developmental netball players when in possession of the ball. | Expert: 6 Developmental: 6 | Notational analysis derived; passes related to game style (defensive pressure, match quarter, decision making complexity), successful passes, causes of errors. | Experts executed more passes under low levels of defensive pressure (63.07 ± 5.72%) *vs.* developmental athletes (47.59 ± 6.49%). Developmental athletes executed a large percentage of passes under high levels of defensive pressure (52.42 ± 6.59%) vs experts (36.94 ± 5.72%).  Developmental athletes executed more passes when only option was available but Experts executed more passes than developmental athletes when there were two ( 37.52 ± 3.02% vs 21.38 ± 5.46%) and three ( 12.24 ± 3.27% vs 1.49 ± 0.52%) options available. Experts executed more successful passes (89.28 ± 3.47%) vs developmental athletes (84.64 ± 2.10%) (p<0.01). |
| Bruce et al. [118] | Elite (professional), 10 teams | N/R | To explore the evolution of team and season performance indicators in elite netball. | 2009 to 2016 seasons, home and away | Nine team performance indicators from commercial provider: centre pass receives, intercepts, deflections, penalties, turnovers, shot attempts, shoot percent, goal assists, rebounds. | Shooting percentage, goal assists, centre pass receives, penalties and turnovers declined from 2009, but began to rise from 2011.  Penalties and turnovers declined from 2015.  Relative similarity shown between each team and season. |
| Croft et al. [117] | Elite (professional) | N/R | To outline the process for capturing, organising and analysing a large performance data-set in professional netball. | 250 | 124 match variables via notational analysis. | 7 netball game style described: safety first, a strong attacking style, another 'safety first' style of playing, reasonably balance style, a low scoring-low loss rate style, a high-risk game style, GA plays a second shooter role. |
| Fox and Bruce [120] | Elite (professional and international) | N/R | To answer a series of questions regarding the implementation and potential use of the two-point rule in Suncorp Super Netball. | 56 Suncorp Super Netball, 36 Fast5 | Possession, activity related to possession, location on court  Shooting statistics: shot made, shot missed, distance from goal. | Suncorp Super Netball: 89% success rate of shots from inner circle, 76% success of shots from outer circles.  Increase in the risk of missing shots as distance from the goal increased.  Fast5: 84% success rate of shots within 3.5m circle, 35% success rate outside 3.5m. |
| McLean et al. [13] | Subject matter experts, n = 11 | N/R | To identify the different components of performance and how they interact to influence performance in netball. | *NR* | Development of a work domain analysis model of netball match-play. | A model of match performance produced showing interrelated objects, processes, functions, values and purposes involved. 5 functional purposes, 19 values and priority measures and  18 general functions deemed necessary for achieving functional purposes.  2 lower levels for the WDA demonstrate object related processes and physical objects. |
| O'Donoghue et al. [28] | Elite | N/R | To determine percentiles for British National Super League netball performances. | 59 (both teams) | Notational analysis derived: Number of centre passes, interceptions, side-lines, backlines, toss ups, defensive rebounds, penalties, turnovers, missed shots, number of goals % of centre passes to goal, turnovers to goal, shots scored. | Top half teams competing against bottom half teams had greater centre passes to goal, number of turnovers, % turnover to goal, % shots score, number of interceptions, number of defensive rebounds and number of goals (p<0.001). |
| *Performance' focused studies* | | | | | | |
| Boothby et al. [122] | Elite (professional) | N/R | To assess the impact of travel, ability and fixture scheduling characteristics on game outcome. | 584 (10 teams) | Performance: Win/Loss and match goal margin Factors: home or away, direction of travel, flight distance, number of days break, previous game location. | Home mean match goal margin higher (p<0.001) than away mean match goal margin (taking into account team ability). Teams travelling west (-6 ± 10) greater disadvantage than for east (-2 ± 10) or north to south (-0 ± 10) travelling (p<0.001) Teams ability accounted for 30% of variance in gaol margin (p<0.001).  Team ability + distance travelled accounted for 32% of variance in match goal margin (p<0.001). |
| Bishop [121] | Elite (professional) | N/R | To assess the influence of travel within and across time zones on netball team performance. | 171 | Combined change in performance with travel assessed by comparing the points difference (home margin - away margin) for each pair of games for each of the four groups of travel. | Points difference: East or west travel > 2 hrs time difference *vs.* local = ES 1.0 large. East or west > 2 hrs time difference vs east or west < 2 hrs time difference = ES 0.5 and vs north or south ES = 0.6.  Points scored at home: no difference between travel groups Points scored away: differences between north or south travel and east or west travel > 2 hrs time difference (p = 0.01. Differences between points scored at home and away for east or west travel > 2 hrs time difference (p =0.01) only. |
| PL = PlayerLoad^TM^, PL2D = PlayerLoad 2 Dimensional, RPE = rating of perceived exertion, RHIE = repeated high intensity effort, N/R = not reported  WA = wing attack, WD = wing defence, GA = goal attack, GS = goal shooter, GD = goal defence, C = centre | | | | | | |

| Supplementary Table S6. Characteristics and key findings of 'nutrition' (n = 3) and training load (n = 4) studies. | | | | | |
| --- | --- | --- | --- | --- | --- |
| Study | Cohort/ sample size (n) | Participant characteristics: age, height, body mass | Purpose | Outcome measures | Key findings |
|  |  |  |  |  |  |
| *Nutrition* | | | | | |
| Broad et al. [6] | International, n = 22 | 18 - 21 yrs 178 ± 5 cm 74 ± 7 kg | To investigate voluntary fluid intake practices and fluid losses during summer and winter conditions in soccer, basketball and netball training and competition. | Fluid intake, body weight changes, sweat loss, dehydration (defined as the body weight loss achieved during the exercise session). | General fluid intake goal for netball players is 600 - 1000 ml/hr. 20L for a squad of 10-12 players should be available for a 2hr training session/match.  Dehydration was higher during summer *vs.* winter training sessions (0.4 ± 0.5 % vs 0.7 ± 0.5 %, p<0.05) and lowest during summer weights sessions *vs.* training and competition (p<0.05). |
| Rumbold et al. [123] | Junior club, n = 11 | 15 ± 1 yrs 166 ± 6 cm 59 ± 7 kg | To examine whether free-living food intake and appetite would be influenced by sport-specific exercise induced EE in young girls. | Estimation of 24 hr EE. Energy intake: self-reported, weighed food diary and 24 hr recall interview Appetite and food parameters (visual analogue scales). | A single intermittent exercise out alters subsequent appetite and energy intake in 13 to 15 yr old girls. 48 hr energy intake was higher following netball exercise *vs.* sedentary period. Girls felt more hungry immediately following netball exercise *vs.* before. |
| Rumbold et al. [124] | School, n = 10 | 15 ± 1 yrs 166 ± 10 cm  53 ± 7 kg | To assess short term acylated ghrelin and test meal energy intake response to acute netball-based exercise and a corresponding sedentary group. | Estimation of 24 hr energy expenditure. Energy intake: self-reported, weighed food diary and 24 hr recall interview Appetite (visual analogue scales) Venous blood samples: acylated ghrelin, insulin and glucose. | 13 to 15 yr old adolescent girls only partially compensated (27%) for the energy expended during netball exercise. Girls felt fuller 20 in into netball exercise *vs.* corresponding time point in the sedentary period. |
| *Training load* | | | | | |
| Brooks et al. [165] | Professional, n = 12 | 26 ± 5 yrs  179 ± 9cm  71 ± 7 kg | To describe the sessional on-court movement demands and external workloads across an elite netball season. | External load: LPS derived distance and acceleration, inertial movement sensors derived PL, PL 2D, jumps. | Distance and PL per minute were lower for training sessions than match-play for all positional groups.  Training: ~41 to 57 m/min, ~6.1 to 7.4 au/min  Match: ~ 58 to 76 m/min, 7.4 to 9.4 au/min  Differences present across session types and with and between positional groups. |
| Chandler et al. [5] | University, n = 8 | 20 (19 to 22) yrs  169 (160 to 177) cm  71 (62 to 81) kg | To investigate the physical demands of different modes of netball training and compare these to match-play. | External workload: Accelerometer derived PL, PL in each axes (vertical, side, forward).  Internal load: Heart rate and RPE. | Skills training had similar PL to match-play, but with lower (p<0.05) mean heart rate. All other training activities (game-based training, traditional conditioning and repeated high intensity effort) had higher PL per minute (p<0.05) but similar heart rate responses. |
| Simpson et al. [166] | Professional, n = 9 | 24 ± 4 yrs 182 ± 8.7 cm 76 ± 11 kg | To examine the relationships between external and internal workload variables in elite female netball over a pre- and competitive-season, with consideration of these relationships relative to playing positions. | External workload: Inertial movement units derived PL, acceleration, deceleration, change of directions, jumps, low- moderate- and high-intensity events. Internal workload: sRPE, summated heart rate zones. | Over the pre- and competition-phases of the season the external workload variables measured are strongly correlated to the internal workload variables. For all positions, except GS the strongest correlation was between PL and sRPE (r = 0.88 to 0.94). For GS the strongest correlation was between the summated heart rate zones and deceleration (r = 0.89). |
| Simpson et al. [164] | Professional, n = 9 | 24 ± 4 yrs  182 ± 9 cm  76 ± 11 kg | To asses and compare external and internal workloads of different netball training drills, compare these to match-play, and assess positional differences. | External load: Inertial movement units derived PL, accelerations, decelerations, change of directions, jumps, low - high intensity events.  Internal load: summated heart rate zones. | PL per minute for conditioning higher than all other drills (p<0.05) (13.13 ± 4.5 vs 4.08 to 8.22 au/min). Conditioning and match-play only training drills that matched or exceed game workloads. |
| EE = energy expenditure, PL = PlayerLoad^TM^, sRPE = session rating of perceived exertion | | | | | |

| Supplementary Table S7. Characteristics, outcome measures and key findings of 'physical qualities' studies (n = 37) | | | | | | | |
| --- | --- | --- | --- | --- | --- | --- | --- |
| Study | Cohort/ sample size (n) | Participant characteristics: age, height, body mass | Validity/ reliability | Comparisons between: | Intervention duration | Outcome measures | Key findings |
|  |  |  |  |  |  |  |  |
| *‘Testing' focused studies* | | | | | | | |
| Barber et al. [138] | *NR,* n = 52 | 24 ± 5 yrs 170 ± 3 cm 65 ± 5 kg | Within- and between-session reliability | N/A | N/A | 5-0-5 test: stationary and flying start. | Within-session reliability: ICC stationary = 0.96 to 0.97, flying = 0.90 to 0.97  Between-session reliability: ICC stationary = 0.97, flying = 0.95.  Learning effect present; day 1 slower than days 3 and 4 for stationary and flying start (p<0.01). |
| Bruce & Moule [139] | Sub-elite, n = 26 | 20 ± 5 yrs 176 ± 6 cm  70 ± 9 kg | Validity | N/A | 6 week | 30-15IFT Criterion validity measure: YoYo IR1). | 30-15IFT is able to detect improvements in performance following a 6 week intervention (pre = 18.6 ± 1.3 *vs.* post = 19.4 ± 1.10, es = 0.7, p <0.01).  Significant relationship with the YoYo IRT1: r = 0.71 to 0.72, p<0.05. |
| Farrow et al. [135] | Elite (highly skilled), sub-elite (moderately skilled), n = 12 Club (less skilled), n = 8 | Elite and sub elite: 16 ± 2 yrs Club: 28 ± 3 yrs | Test re-test reliability Discriminant validity | Playing levels or standards | N/A | Reactive agility test: shuffle time, sprint time, total time, decision time and movement detection accuracy.  Planned test: Shuffle time, sprint time, total time. | Test re-test reliability: reactive ICC = 0.83, planned ICC = 0.81 Reactive sprint time faster for highly skilled (1.07 ± 0.07s) and moderately skilled (1.12 ± 0.11 s) *vs.* less skilled (1.23 ± 0.06, p<0.05), but no significant difference in planned sprint time.  Highly skilled players have a quicker total time (3.49 ± 0.22 s) *vs.* less skilled (3.65 ± 0.18s) players in the planned test, p<0.05). Both highly (3.57 ± 0.14 s) and moderately (3.60 ± 0.09s ) skilled players have a faster time than the less skilled (3.83 ± 0.11) in the reactive test (p<0.05). |
| Gasston & Simpson [140] | Reliability assessment, n = 4  Validity assessment n = 20 | N/R | Test re-test reliability  Construct validity | Standards | N/A | Netball Specific Fitness Test score. | Three subjects differed by 2 stages or less on re-test. No positional differences identified. Median performance for higher standard (Level 8, stage 5) greater than lower standard (Level 7, stage 6) (p>0.05). |
| Humphries et al. [136] | A-grade, n = 25 | 18 to 39 yrs | Within session reliability Validity | N/A | 6 week | Single leg hop jump onto a force-plate: maximal displacement, peak GRF in mediolateral, anteroposterior and vertical components. | Increases in displacement (ES = 0.63 to 0.76, p =0.00), and mediolateral and anteroposterior forces (es = 2.76 to 3.00, p = 0.00) detected following 6 weeks of training. Excellent with-session reliability at baseline and at 6-weeks for displacement (ICC = 0.92 to 0.97). |
| Mungovan et al. [134] | Elite, n =18 | 24 ± 5 yrs 183 ± 6 cm 78 ± 16 kg | Test re-test reliability Criterion validity | N/A | N/A | The Net Test Performance measures: time for each component Physiological measures: RPE, mean absolute and relative HR, peak absolute and relative HR Criterion validity measures: vertical jump height, 5-, 10-, 20-m sprint times, 5-0-5 test, YoYo IR1. | Test re-test reliability:  No difference in total time between trials, ICC = 0.90, CV = 1.7%, TEM = 0.69 Other performance measures: ICC = 0.37 to 0.93, TEM = 0.05 to 0.69, CV = 1.7 to 7.9% Physiological measures: ICC = 0.04 to 0.73, TEM = 0.75 to 5.01, CV = 2.8 to 9.5% *Very large* relationships (p<0.05) between total time and vertical jump (*r* = -0.77), 5m sprint (*r* = 0.70), 10 m sprint (*r* = 0.72), 20m sprint (r = 0.75), 5-0-5 test (*r* = 0.80) and a *large* relationship with YoYo IR1 derived VO_2_max (*r* = -0.66, p<0.05). |
| Reid et al. [137] | School, n = 40 | 16 ± 1 yrs 170 ± 8 cm 63 ± 7 kg | Inter- and intra-rater reliability | N/A | N/A | Netball movement screen tool: movement competency screen, jump tests, star execution balance, active straight leg raise. | Inter-rater reliability: ICC scores = 0.65 to 0.99. No significant difference between mean scores of rater’s. Intra-rater reliability: ICC scores = 0.77 to 0.96. No significant difference in mean scores between time points. Substantial to poor inter and intra-rater reliability for individual movement competency screen test scores. |
| Scanlan et al. [133] | Elite: international, n = 7, national n = 7 | International: 28 ± 4 yrs, 76 ± 9 kg National: 20 ± 2 yrs, 81 ± 21 kg | Discriminant validity | N/A | N/A | The Net Test Performance measures: time for each component Physiological measures: RPE, mean absolute and relative HR, peak absolute and relative HR. | Total time: International = 40.56 ± 1.85 s, national = 42.77 ± 2.13 s. International *very likely* faster vs national players (es = 1.11). Defensive shuffle *very likely* faster for International vs national players (es = 2.49, p<0.01), left side step shuffle *likely* faster and dodge *possibly* faster for international *vs.* national. All other components had similar performance times.  *Unclear* differences in physiological measures. |
| *‘Anthropometric' focused studies* | | | | | | | |
| Chang et al. [149] | State, n = 14 | 21 ± 3 yrs 175 ± 6 cm 70 ± 8 kg | N/A | Other sport (golf) and controls | N/A | BMD: total-body, lumbar spine, bilateral proximal femur and forearm. Fat mass, fat free soft tissue mass, and fat percentages. Muscle performance measures: grip strength and trunk extensor endurance (Sorensen test). | Total body BMD = 1.24 ± 0.7 Body fat (%) = 27.7 ± 4.6 Differences observed between sports and sedentary controls: netballers taller and greater lean mass than control and golfers, with greater BMD, grip strength and body mass *vs.* control (p<0.05). |
| Egan et al. [148] | University and club, n = 20 | 21 ± 1 yrs 168 ± 7 cm 64 ± 7 kg | N/A | Other sport (rugby union, distance running) and controls | N/A | BMD: total-body, lumber spine and left proximal femur. Fat mass, fat free soft tissue mass, and fat percentages. | Total body BMD = 1.18 ± 0.0 Body fat (%) = 26.3 ± 3.7 Netball has positive effects on BMD *vs.* sedentary control individuals. |
| Soh et al. [49] | International: senior n = 12, reserves n = 11, junior n = 9 | 18 ± 4 yrs 171 ± 5 cm 64 ± 7 | N/A | Playing levels/standards | N/A | Height, body mass, body fat percentage (from sum of 7 skinfolds predicted), body somatype. | Height (cm): 171.75 ± 4.33, junior = 168.50, ± 5.70, reserves = 171.64 ± 3.67. Body fat (%): elite = 25.8 ± 4.59, junior = 23.69 ± 6.72, reserves = 23.69 ± 4.36. No differences observed between playing levels (p>0.05). |
| Soh et al. [51] | School national tournament: good n = 50, medium n = 50m fair n = 50 | 17 ± 1 yrs 165 ± 6 cm 59 ± 10 kg | N/A | Playing levels/standards | N/A | Height, body mass, body fat percentage (from sum of 4 skinfolds predicted), body somatype. | Height (cm): good = 166.82 ± 6.10 , medium = 164.49 ± 6.11, fair = 162.97 ± 6.49. Body fat (%): good = 12.92 ± 3.87, medium 12.10 ± 3.44, fair 11.66 ± 3.07. Height is the only difference identified between playing levels (good *vs.* fair players, p<0.01). |
| *‘Physical characteristics' focused studies* | | | | | | | |
| Bock-Jonathon et al. [146] | University: Group A (2nd team), n = 5, Group B (3rd and 4th teams) n = 12, Group C (5th and 6th teams) n = 13 | N/R | N/A | Playing levels/standards | N/A | Technical performance: netball agility test, passing accuracy, repeated passing test, pivot and pass test. Tactical knowledge test. | Significant differences between groups (i.e., playing standards. Group A had a greater standard score mean for technical performance compared to groups B and C (p<0.05), and Group B had a greater score *vs.* group C (p<0.05). Netball agility test (s): group A = 17.68 ± 0.28, Group B = 17.9 ± 0.75, Group C 18.62 ± 0.60, groups A and B faster than group C (p<0.05). |
| Clark et al. [141] | Club, n = 23 | 29 ± 6 yrs 172 ± 7 cm 68 ± 10 kg | N/A | Limbs | N/A | Leg length, barefoot eyes close balance test, triple hop, single hop, vertical hop. | Clinically significant (i.e., >10%) asymmetries for the eyes closed balance test (43.7 ± 24.0 %) and vertical hop (13.1 ± 12.8 %). *Small* difference between limbs on vertical when normalised for leg length (es = 0.18). |
| Dos’Santos et al. [142] | NR, n = 21 | 18 ± 1 yrs 174 ± 6 cm 67 ± 5 Kg | N/A | Between limbs | N/A | 5-0-5 test and COD deficit, 10-m sprint time. | 10m sprint time = 1.97 ± 0.06 s 5-0-5 left = 2.52 ± 0.08 s, right = 2.50 ± 0.10 s Significant imbalance between dominant and non-dominant limbs for 5-0-5 test (-2.2 ± 2.0 %, p<0.001, ES = -0.62) and COD deficit (-11.1 ± 10.1%, p<0.001, ES = -0.62). |
| Ferreira et al. [143] | Club, n = 25 | 174 cm 68 Kg | N/A | Over time: pre and post season | N/A | Anthropometrics: sum of 6 skinfold (body fat %), stature and body mass. Physical: Illinois agility test, computerised balance test and vertical jump. Biomechanical assessment: symmetry, dynamic mobility and local stability in different zones; limb-pelvic region, hip girdle, lower limb and neurodynamic Injuries monitored for: mechanism, diagnoses, severity, type, time off training. | Body mass (~68 vs. 70 kg), BMI (~22 *vs.* 23) and body fat % (27 *vs.* 28%) all increased across the season (p<0.05, ES = 0.23 to 0.44). Increases in agility and balance performance were observed (p<0.01 ES = 0.72 to 1.10), but decreases in explosive power. Only 5 out of the 38 biomechanical assessment outcomes changes across the season. |
| Graham et al. [144] | State level, n = 46 | 20 ± 4 yrs | N/A | Positions | N/A | Stature, single and triple single-leg bound (take off one foot, land two), CMJ, 5-, 10-, and 20-m sprint speed, 5-0-5 test, YoYo IR Level 1. | Differences are present the physical qualities across positional groups. The largest difference in physical qualities was in stature between midcourt athletes (172 ± 4 cm) and defenders (181 ± 2 cm, es = -2.58) and shooters (178 ± 6 cm, es = -1.26). Midcourt players had the greatest sprint and COD ability and triple hop distance, but defenders had the greatest CMJ height (52 ± 5 cm *vs.* shooters 47 ± 7 cm, midcourt = 48 ± 4 cm, es = 0.56 to 0.61). |
| McKenzie et al. [125] | Youth (School): grade 1 n = 26, grade 2 n = 42, grade 4 n = 8 | 13 ± 1 yrs 167 ± 6 cm 61 ± 13 kg | N/A | Playing levels/standards and positions | N/A | Modified Start Excursion Test (SEBT), 2-, 5-, 10-, 15-, 20-m sprint times, T-test, vertical and horizontal jump distance, prone hold, time to stabilisation. | Differences are present the physical qualities across playing levels. Grade 1 players have a *very likely* greater prone hold (94 ± 4 s *vs.* 53 ± 26 s, ES = 1.17) and T-test (13 ± 1 s *vs.* 15 ± 1 s, ES = 1.31) ability, and *likely* greater 15-m sprint (3.11 ± 0.16 *vs.* 3.27 ± 0.21 s, ES = 0.61) , 20-m sprint (3.64 ± 0.25 *vs.* 3.91 ± 0.38 s, ES = 0.69), vertical jump (40 ± 8 cm *vs.* 35 ± 5 cm, ES = 0.61), and time to stabilisation ability (1026 ± 279 ms vs. 1306 ± 424 ms, ES = 0.69), *vs.* Grade 4 players.  Positional differences were playing level dependent; Grade 1 players had *unclear* differences between positions for all tests except 20-m sprint speed. Grade 2 players had *possibly* to *likely* differences between positions. |
| Pruyn et al. [132] | Elite n = 9, sub-elite n = 17, representative n = 11 and recreational n = 8 | 24 ± 4 yrs 72 ± 8 kg 175 ± 7 cm | N/A | Playing levels/standards | N/A | Stature, body mass, vertical stiffness, passive stiffness of lateral gastrocnemius , medial gastrocnemius, soleus , Achilles aponeurosis in 2 positions (lying and standing). | Elite players were significantly taller than recreation players (180 ± 5 cm *vs.* 171 ± 6 cm, p = 0.018) and had significantly higher vertical stiffness (220 ± 42 n^-1^·m^-1^·kg^-1^ *vs.* 173 ± 43 n^-1^·m^-1^·kg^-1^, p = 0.018, *large*). A *large* difference in vertical stiffness was present between elite players and sub elite (184 ± 29) and representative (182 ± 25).  Elite players demonstrate superior passive stiffness measures compared to the lower playing standers, with *moderate* to *large* positive differences. |
| Simpson et al. [18] | Part A: Elite n = 149, sub-elite n = 115, regional n = 126, State level: U21 n = 71, U19 n = 173, U17 n = 210  Part B: Elite n = 28, subelite n = 31 | NR | N/A | Part A: player levels/standards  Part B: over time | Part B: 4 years | Anthropometrics: stature and body mass. bone density, lean mass and body fat % (subsample of elite and sub elite only). Performance: vertical jump, 5-, 10-, 20m sprint time, YoYo IR Level 1. | Part A: Elite players were significantly older (24 ± 3 yrs) , taller (182 ± 7 cm), heavier (73 ± 7 kg), had a higher vertical jump (52 ± 1 cm) and faster 20-m sprint speed (3.32 ± 0.11 s) compared to all other standards (p<0.001, ES = 0.40 to 0.93), and a higher YoYoIRT1 test score (1251 ± 72 cm) *vs* all standards except U21s (p< 0.05, ES = 0.32 to 0.74). U17s had a faster 5-m sprint speed (1.12 ± 0.07 s) compared to the elite, sub-elite and U19 players (p<0.05, ES = 0.36 to 0.58). Part B: Changes in physical profiles are apparent over time. Vertical jump increased from 2014 to 2017 (p<0.01, ES = 1.18) and YoYo IRT1 distance was higher in-season than pre-season (p<0.05, es = 0.17), with *very likely* increases observed from 2014 to 2017 (ES = 0.62). |
| Sinclair et al. [147] | State, n = 77 | 18 ± 2 yrs  179 ± 8 cm  70 ± 11 kg | N/A | Age group (U18, U19, U21)  Positions | N/A | Body mass, stature, skinfolds, star excursion balance test, standing broad jump, double and single leg vertical jump, horizontal pull, press ups, prone bridge, YoYo IR1, Octorepeater test, 5-, 10-. 40-m speed. | Height and body mass increased with age (p<0.05). GS tallest (181 ± 6 cm) position. No significant difference in muscular endurance tests or vertical jumps between age groups. YoYo score increased with age groups (p<0.05). Senior players had the highest fatigue index (8.5%) (p<0.05). |
| Soh et al. [50] | Milo Malaysian-Singapore Series Games n = 52 | NR | N/A | Positions | N/A | VO2max (estimated from 12 minute run), anaerobic performance (8 x 35 metre runs), leg extension and flexion strength test, SEMO agility test, vertical jumps, 5-, 10- and 25-m sprint time. | Significant (p<0.05) differences in leg extensor strength between positions: defenders had great right (169.33 ± 26.94 N·m^-1^) and left (162.86 ± 28.65 N·m^-1^) leg extensor strength *vs.* centres (right = 44.86 ± 14.49 N·m^-1^, left = 133.93 ± 15.98 N·m^-1^). No other significant positional differences were present. |
| Soper et al. [131] | Elite | 19 ± 4 yrs 177 ± 8 cm 70 ± 11 kg | N/A | Hypermobile and non-hypermobile | N/A | Self-reported questionnaire: netball experience, playing position, injury history, hypermobility, dysautonomiac symptoms. Beighton score (hypermobility), single leg stance and posturography, star execusion balance test. | 63% of players had general joint hypermobility (n = 17), 15% had joint hypermobility syndrome (n = 4). Distinctly hypermobile athletes had greater posturographic values for path area and velocity on the left side (p=0.002), demonstrating increased postural instability. |
| Thomas et al. [16] | Academy, n = 43 | 16 ± 1 yrs 174 ± 6 cm 67 ± 8 kg | N/A | Positions | N/A | Stature, body mass, single leg hop, vertical jump (squat jump and counter movement jump), 5 and 10m sprint time, 5-0-5 test, 30-15IFT. | Differences are present in the physical profiles of players between positional groups. Defenders are taller (177 ± 5 cm) and heavier (71 ± 10 Kg) than centres (p<0.05, ES = 1.1 to 1.6) but centres have a greater SJ (41 ± 5 cm), CMJ (42 ± 4 cm), 5-0-5 ability (right = 2.41 ± 0.06 s, left = 2.44 ± 0.11 s), and 30-15IFT score (18.5 ± 1.3 km·h^-1^) (p<0.05, ES = 1.0 to 1.4). Centres (1.12 ± 0.6 s) and defenders (1.10 ± 0.09 s) had greater 5-m sprint ability *vs.* shooters (1.18 ± 0.05 s, p<0.05, ES = 0.9 to 1.0). |
| Thomas et al. [20] | N/R, n = 21 | 174 ± 6 cm  67 ± 5 kg | N/A | Other sports | N/A | CMJ concentric peak force, IMTP peak force, dynamic strength index (= CMJ-PF/ IMTP-PF). | Dynamic strength index = 0.89 ± 0.22, no differences between sports CMJ-PF = 1651 ± 239 N, greater than female cricket and soccer (p<0.05, ES = 0.61 to 0.86) IMTP-PF = 1925 ± 374 N, greater than female cricket (p<0.05, ES -0.74). |
| Thomas et al. [145] | Youth academy, n = 26 | 16 ± 1 yrs 174 ± 6 cm 66 ± 7 kg | N/A | Relationship: maximal isometric strength, vertical jump, sprint and change of direction speed | N/A | IMTP peak force, vertical jump height (squat jump and counter movement jump), 5 and 10 m sprint time, 5-0-5 test. | Relative isometric strength (IMTP-PF) moderate to strong (*r* = -0.48 to -0.66) correlation with 5-0-5 times. Vertical jump performance large to very large (*r* =-0.60 to -0.71) correlations with 5-0-5 times. Stronger players (top 13 IMTP) were faster over 5-m (p<0.05, ES = 1.1) and 10-m ( p<0.05, ES = 1.2), had a quicker 5-0-5 time (ES = 1.2 to 1.7), and greater vertical jump performance (es = 0.9 to 1.0). |
| Venter et al. [130] | Elite, n = 20 | 20 ± 2 yrs 177 ± 5 cm 70 ± 7 kg | N/A | Relationship: FMs and performance tests | N/A | FMS, CMJ, 5- and 10-m sprint times, repeated sprints. | Correlations between rotary stability and vertical jump (*r* = 0.54, p = 0.04), FMS total and 5-0-5 time (*r* = -0.56, p = 0.02), trunk stability and 5-0-5 time (*r* = -0.52, p=0.03), FMS total and repeat sprints (*r* = 0.51, p =0.03), trunk stability and repeat sprints (*r* = 0.50, p = 0.04). |
| Withers & Roberts [129] | International, n = 5 | 21 ± 2 yrs 175 ± 3 cm 74 ± 6 kg | N/A | Other sports | N/A | Maximal oxygen uptake and maximum anaerobic power (treadmill). Anthropometrics: height, body mass and % body fat from sum of 4 skinfolds. | VO_2_max = 44.8 ± 1.2 ml^-1^·kg^-1^·min^-1^, maximum power output = 953.0 W, vertical velocity = 1.31 ± 0.11 m·s^-1^, % body fat = 28.9%. Netballers were the heaviest but with the highest power output. |
| *Intervention' focused studies* | | | | | | | |
| Barnes et al. [153] | Univeristy, n = 19 | ~ 19 yrs | N/A | N/A | 6 week: core stability, gluteus medius strengthening and proprioceptive balance exercises | Star excursion balance test. | Improvements in average distance in the anterior, medial, posterior and lateral reach directions post intervention (p<0.05). |
| Bell et al. [128] | International, n = 21 (n = 10 for intervention) | 18 ± 1 yrs | N/A | Positions | 4 month: aerobic and anaerobic work, weight training, circuit training, plyometrics, and calisthenics | Anthropometrics: stature and body mass, sum of 4 skinfolds. Forced vital capacity. Performance: VO_2_max, Wingate test (peak power output, end power output, mean power output). | Positional differences in the physical profiles of players. Defenders and attackers are taller (p<0.001), heavier (p<0.05) and have a greater forced vital capacity (p<0.05) compared to centres. No difference in performance measures.  Following the training programme significant increases in height (p<0.01), body mass (p<0.05), forced vital capacity (p<0.01) and anaerobic performance (p<0.01). |
| de Villiers et al. [150] | University, n = 20 | Control group: 20 ± 1 yrs, 170 ± 6 cm, 68 ± 8 kg Barefoot: 20 ± 2 yrs, 170 ± 6 cm, 68 ± 8 kg | N/A | N/A | 8 week: barefoot training *vs.* normal netball shoes | Anthropometrics, 10 - and 20-m sprint, 5-0-5 test, the Athlete Single Leg Balance tests: overall stability index, anterior-posterior stability index, medial-lateral stability index. | Barefoot group: significant and *small* to *large* increases in 5-0-5 test, overall stability, anterior-posterior and medial-lateral stability of the right leg from pre to post. Small increases in the stability markers for the left leg were observed.  Control group: no significant differences in testing scores pre and post training, but moderate increases in overall stability for the right leg, anterior-posterior stability of the right leg and small increases in stability of left leg and agility to the right. |
| Hopper et al. [126] | Junior, n = 23 (control group n = 10, experimental group n = 13) | 12 ± 1 yrs 163 ± 8 cm 52 ± 9 kg | N/A | N/A | 6 week: neuromuscular training programme | Netball movement screening tool Neuromuscular performance test: CMJ, 5-, 10-, 20-m sprint speed, 5-0-5 test. | Neuromuscular training group saw *large* increases in 10- and 20-m sprint performance, 5-0-5 performance, CMJ jump height and peak power, and netball movement screen total score (p<0.05, es > 0.8), with greater changes scores compared to the control group (p<0.05). |
| Manimmanakorn et al. [151] | Well-trained, n = 30 | 20 ± 3 yrs 168 ± 7 cm 65 ± 7 kg | N/A | N/A | 5 weeks: control *vs.* restricted blood flow (Kaatsu training) *vs.* hypoxic training | Muscular performance: MVC 3 seconds, fatigue during MVC 30 seconds, number of repetitions at 20% 1 repetition maximum load Sport specific tests: CMJ, 5- and 10-m speed, 5-0-5 test and maximal multistage 20-m shuttle test predicted VO_2_max. Visual analogue scale for determining knee flexor and extensor muscle pain MRI scan to determine cross sectional area of knee flexor and extensor muscles. | *Substantial* differences in improvements in muscular performance measure between groups. Percentage change from baseline for MVC 3 seconds (15 ± 11 %), MVC 30 seconds (17 ± 18%) greatest for HT group, but change in number of repetitions at 20% 1 repetition maximum load greatest for KT group (140 ± 34%).  Some differences in the change in performance of sport specific test were present*: likely* differences in 5-0-5 performance and VO_2_max changes between HT and control group, *very likely* differences in 5-0-5 test between KT and HT. Combined cross sectional area of extensor and flexor muscles increased by 6.6 ± 4.5 % KT training, 6.1 ± 5.1 % HT training, 2.9 ± 2.7% control training. |
| McKenzie et al. [4] | School, n = 81 (control group n = 36, intervention group n = 45) | Control group: 13 ± 1 yrs, 169 ± 4 cm, 65 ± 10 kg Intervention: 13 ± 1 yrs, 165 ± 6 cm, 57 ± 10 kg | N/A | N/A | 7 weeks: netball dynamic warm up *vs.* traditional warm up | Y balance test, 2-, 5-, 10-, 20-m sprint speed, vertical and horizontal jump performance, prone hold, time-to-stabilisation, T-test. | Players following the netball dynamic warm up had increases in prone hold (p = 0.004, ES = 0.48), 20-m sprint time (p = 0.001, ES = 0.42), vertical jump (p = 0.001, ES = 0.53) and Y balance test (p = 0.003, ES = 0.34). Prone hold and vertical jump height improved more in intervention group than control (p<0.01). Horizonal jump distance decreased in intervention compared to control (p=0.03). |
| McKeown et al. [127] | Elite, n = 12 | 20 ± 0.4 yrs 182 ± 54 cm 74 ± 7 kg | N/A | N/A | 18 weeks | CMJ with and without 15 Kg load (peak velocity, mean power, jump height), drop jump (jump height, contact time, reactive strength index). | *Large* improvements in CMJ power (24%, ES = 1.45) and velocity (12%, es = 1.13), and loaded CMJ power (19%, ES = 1.49). *Moderate* improvements in loaded CMJ velocity (8.4%, ES = 1.01) and drop jump reactive strength index (35%, ES = 0.97). S*mall* improvements in loaded CMJ jump height (11%, es = 0.59) and drop jump height (10%, es = 0.52).  For all three jumps most of the improvements occurred within the first 7 weeks, with minimal further improvements in the remainder of training period. |
| Roopchand-Martin & Leu-Chin [48] | Elite and sub-elite club, n = 26 | 19 ± 2 yrs | N/A | N/A | 3 weeks: plyometric training | Vertical jump, standing broad jump, Illinois agility test. | All performance measures increase from pre to post training. Vertical jump increased by 1.81 ± 2.74 cm (p = 0.023), broad jump increased by 12.04 ± 12.11 cm (p = 0.002), Illinois agility time decreased by 0.45 ± 0.79 s (p = 0.045). |
| Venter et al. [152] | Well-trained club, n = 17 (backward group n = 10, forward group n = 7) | 20 ± 1 yrs | N/A | N/A | 6 weeks: forward *vs.* backward running | 5-, 10- 20-m sprint speed, 5-0-5 test, T-test, ladder test and vertical jump. | Backward running group had greater changes in 5-0-5 test time, T-test and ladder test than the forward group (p<0.05). No significant differences observed in vertical jump performance observed for either group. |
| N/A = not applicable, N/R = not reported, ICC = intra-class correlation coefficient, 30-15IFT = 30-15 intermittent fitness test, ES = effect size, CV = coefficient of variation, TEM = typical error of the mean, *r* = correlation coefficient, VO_2_max = maximal oxygen uptake, RPE = rating of perceived exertion, HR = heart rate, BMD = bone mineral density, COD = change of direction, CMJ = counter movement jump, IMTP = isometric mid-thigh pull, PF = peak force, FMS = functional movement screen, KT = Kaatsu training HT = hypoxic training | | | | | | | |

| Supplementary Table S8. Characteristics and key findings of 'psychology' studies (n = 13) | | | | | |
| --- | --- | --- | --- | --- | --- |
| Study | Cohort/ sample size (n) | Participant characteristics: age, height, body mass | Purpose | Outcome measures | Key findings |
|  |  |  |  |  |  |
| Devonport et al. [159] | U17 & U19 International, n = 33 | 17 ± 1 yrs | To explore the stressors and personal and situational factors influencing stressor appraisals experienced by junior national netball players and how these athletes cope with such stressors. | Semi-structured interviews and focus group interview derived themes and codes.  Stressors coded under four sources: acute, chronic, expected and unexpected. | Players articulated a number of stressors for each source (acute, chronic, expected and unexpected) resulting from netball but also academic, work, social and developmental factors.  The utilisation of future-oriented coping strategies were associated with successfully managing multiple stressors and a sense of well-being. |
| Edwards et al. [158] | University, n = 45 | 22 ± 2 yrs | To examine the relative influence that directional perceptions and intensity of anxiety symptoms have upon performance and to examine the interactive effects of anxiety intensity on sub-components of performance. | Modified Competitive State Anxiety Inventory-2 Physiological arousal (heart rate) Subjective self-assessment of performance. | An interaction (p<0.05) emerged between the two factor cognitive anxiety and physiological arousal quadrant analysis.  With low physiological arousal, netballers with high cognitive anxiety performed better than those with low cognitive anxiety. |
| Grobbelaar et al. [162] | Provincial: U19, n = 81 U21, n = 63 Senior, n = 41 | 21 ± 4 yrs | To determine whether the psychological skill levels of South African provincial netball players in different playing positions. | Athletic Coping Skills Inventory: seven psychological skills and attributes. | Goal attack and wing defence players consistently outperformed other positional groups and the goal shooters showed the lowest psychological skill levels. |
| Kruger et al. [163] | University, n = 18 |  | Use the Bulls Mental Skills questionnaire to develop preliminary South African university norms for sport codes | Bulls Mental Skills questionnaire: seven subscales. | Netball total score = 124.17 ± 17.57. |
| leCouteur et al. [22] | Elite |  | To identify recurrent interactional practices in the data and describe patterns of association between such practices and defensive outcomes in netball. | Descriptive and conversation analysis of defensive match-play. | Higher frequency communication between defenders when opposition players successful obtained shots at goal. Uptake of communication was dependent upon speakers taking account in their verbal and non-verbal conduct, of both their team mates current orientation and visual access to the defensive problem. |
| Palmer et al. [160] | International U21, n = 17 | 18 ± 1 yrs | To evaluate an intervention based on the predictions of Massux's revised Theory of Planned Behaviour designed to improve fitness training adherence in elite netball players. | Training behaviour and adherence: self-report training diary.  Revised theory of planned behaviour measures via questionnaire: intention, self-efficacy, attitude toward current and new behaviours, perceived vulnerability, perceived social norms, intervention check. | The use of the revised Theory of Planned behaviour to enhance fitness training adherence in junior elite netball players supported. Large changes in training adherence from baseline to post intervention for 76% (n = 13) of players (es = 0.94 to 3.81). Changes in post intervention 'perceived vulnerability' and 'attitude towards current behaviour' suggesting that cognitive changes have occurred. |
| Terry et al. [161] | Club, n = 162 | 30 ± 6 yrs | To investigate perceptions of cohesion and mood among rowers netball and rugby teams. | Group environment questionnaire and profile of mood states. | The perceptions of cohesion predicted 1.50 to 9.00% of the variance in mood responses. Perceptions of the degree of collective integration around social aspects of the team were unrelated to any of the positive and negative mood states. Netball players personal feelings of attraction to the teams task were inversely related to feelings of tension and anger. |
| Wakefield & Smith [7] | University, n = 32 | 20 ± 2 yrs | To test the effects of different frequencies of PETTLEP imagery on a specific motor task. | Pre and post intervention: Movement imagery questionnaire Performance test (shooting task, 100 shots). | PETTLEP imagery 3x week improved performance measures (p<0.05), whereas the lower frequency and control did not. Imagery 3 x a week reported the most positive thoughts about the usefulness of the imagery. |
| *Motor Learning and Decision Making* | | | | | |
| Bishop et al. [121] | International, n = 13 | 20 ± 3 yrs | To examine the contribution of eye movements to netballers decision-making. | SR Research EyeLink 100 eye tracker derived eye movements: percentage dwell time, number of fixations, initial saccade latency. | Spatially invalid auditory cues reduced performance accuracy relative to valid ones. Semantically invalid cues increased response times relative to valid ones. No accompanying changes in visual attention. |
| Bruce et al. [155] | International open age (expert), n = 19 International U17 (developmental), n = 20 Club (lesser skilled), n = 19 | Expert: 24 ± 5 yrs Developmental: 17 ± 5 yrs Less skilled: 28 ± 5 yrs | To determine how the development of motor skill execution may influence both perceptual-cognitive and perceptual-motor decision making performance across a development spectrum. | Motor skill execution test: execution and overall accuracy. Perceptual-cognitive decision making task: decision response accuracy, visual search rate, location accuracy  On court perceptual motor decision making task: decision and execution response accuracy. | Performance on motor skill-execution test directly related to expertise; experts had greater passing accuracy than development athletes, who in turn were more accurate than lesser-skilled (p<0.01). Decisions requiring movements (perceptual motor) elicited more accurate decision making vs. simple verbal responses (perceptual cognitive) irrespective of skill level. Expert were better able to distinguish between and appropriately response to scenarios of differing lengths, namely long and short passing options. |
| Bruce et al. [156] | International (skilled), n = 28 Club (novice), n = 16 | Skilled: 21 ± 4 yrs Novice: 25 ± 3 yrs | To examine the scope of domain specificity within netball. | Response accuracy during difference playing scenarios (decision making tasks). | Skilled goalers and centre court players were more accurate than the novice players indicating decision making is a key discriminator between skilled and lesser skilled performers.  Limited evidence to support position specificity in perceptual-cognitive skills. |
| Farrow [157] | International:  Open, n = 17 U21, n = 25 U19, n = 15 U17 n = 16 | Open: 25 ± 5 yrs U21: 19 ± 1 U19: 18 ± 1 U17: 17 ± 1 | To examine the development of perceptual-cognitive and perceptual-motor skill in highly skilled netball players. | Perceptual cognitive tests: pattern recall and decision-making Perceptual motor tests: passing skill under single and dual task load, and reactive agility. | Pattern recall, decision making accuracy and passing skill explained the greatest amount of variability between groups (77.6%) and could successfully distinguish the open squad from the other squads and the U21 squad from the U19 and U17 squads. |
| Richards et al. [21] | Elite netball team and coaches |  | To explore the complexity of developing decision-making skills in elite netball, through 5 stages: 1. development of coaches situational framework for an attacking centre pass 2. developing a team decision-making framework with players 3. Developing on court decision making in players 4. maintaining decision making 5. Application and transfer to world stage. | Stage 1: establishment of shared vision of what centre pass should look like Stage 2:theory sessions and practical/match sessions facilitating development of the shared vision Stage 3: reflective activities and notational analysis Stage 4: reflective activities and notational analysis Stage 5: reflective activities and notational analysis | Demonstrates how a coach can empower players individually and collectively as a team to reflect 'on action', facilitating the coach, players and team 'reflection-for-action' and ultimately 'in-action'. |

Declarations

**Acknowledgements:** There are no acknowledgements.

**Funding:** The authors declare no specific grant for this research from any funding agency in the public, commercial or non-for-profit sectors.

**Conflict of interests:** Sarah Whitehead, Jonathon Weakley, Stuart Cormack, Helen Alfano, Jim Kerss, Mitch Mooney and Ben Jones declare that they have no conflicts of interest relevant to the content of this review.

**Availability of data and material:** Data supporting this review are available from the corresponding author upon request.

**Code availability:** Not applicable.

**Author contributions:** SW and BJ conceptualised the review. SW led the searching, study section and data charting with JW and BJ. SW, BJ, JW and SC led the writing of the manuscript, with HA, JK and MM contributing significantly to the gaps in the literature and future directions. All authors critically revised the manuscript. All authors read and approved the final manuscript.
